# Preliminary Analysis of the Proportion and Characteristics of Dual BCR B Cells in SLE Model Mice and Patients via scRNA-Seq Combined with scBCR-Seq Technology

**DOI:** 10.3390/cells15100914

**Published:** 2026-05-17

**Authors:** Kai Quan, Hongxia Yang, Guangtian Tang, Ziwei Li, Hailin Zou, Jing Ma, Xinsheng Yao

**Affiliations:** 1 Department of Immunology, Zunyi Medical University, Zunyi 563000, China; m19191301316@163.com (K.Q.); yanghongxiafly@126.com (H.Y.); 13678401091@163.com (G.T.); lzwsxk@126.com (Z.L.); 15811672460@163.com (H.Z.); keewood@126.com (J.M.); 2 Guizhou Provincial Research Center for Applied Immunomolecular Engineering, Zunyi Medical University, Zunyi 563000, China; 3 Guizhou Provincial Innovation Base for Graduate Education in Immunology, Zunyi Medical University, Zunyi 563000, China; 4 Guizhou Provincial Key Discipline of Immunology, Zunyi Medical University, Zunyi 563000, China

**Keywords:** systemic lupus erythematosus (SLE), BCR CDR3 repertoire, dual BCR B cells, single-cell RNA sequencing, single-cell VDJ (BCR) sequencing

## Abstract

Systemic lupus erythematosus (SLE) is an autoimmune disease characterized by immune dysregulation and multi-organ damage. Abnormal B cell activation and autoantibody production constitute the core pathological mechanism of SLE. However, the proportion, BCR pairing types, clonal evolution patterns, and transcriptomic features of dual BCR B cells in SLE remain incompletely elucidated. In this study, we employed single-cell RNA sequencing (scRNA-seq) combined with single-cell B cell receptor repertoire sequencing (scBCR-seq) to preliminarily analyze the proportion and characteristics of dual BCR B cells in SLE model mice (MRL/Lpr and SLE.Yaa) as well as in peripheral blood from SLE patients. The results showed: (1) Compared with control groups, the proportion of dual BCR B cells in SLE model mice and patients exhibited a decreasing trend, whereas the diversity of the CDR3 repertoire decreased and clonality increased. Increased clonal sharing was observed between single BCR B cells and dual BCR B cells. The main pairing types of dual BCR B cells were H + κ1 + κ2, H1 + H2 + κ, and H1 + H2 + κ + λ, with preferential utilization of autoimmunity-associated V gene families such as *IGHV4-34*, and high expression of IGHG subtypes. (2) Tracking analysis of B cell receptor clonality and effector molecule expression revealed that in SLE, dual BCR B cells tend to enrich in IFN-α/γ responses, TNF-NFκB inflammation, and complement pathways, and highly express interferon-related genes such as *Ly6a*, *Isg15*, *MX1*, and *IFI6*. (3) In both single BCR B and dual BCR B cells from SLE patients, the proportion of the naïve B cell subset decreased, whereas the proportions of plasma and Breg subsets increased and exhibited clonal expansion. SLE dual BCR Breg cells highly expressed *IL10*, *HSPA1A*, and others. This study is the first to reveal, at the high-throughput single-B-cell level, that the proportion, subset origin distribution, CDR3 repertoire composition, and effector molecule expression of dual BCR B cells display unique characteristics in SLE model mice and patients, providing baseline comparative data and novel research perspectives for further investigation into B cell effector functions and mechanisms in SLE patients.

## 1. Introduction

SLE is an autoimmune disease characterized by immune dysregulation and multi-organ damage. Its core pathological feature is the breakdown of immune tolerance to self-antigens, manifested by excessive T cell activation, abnormal B cell proliferation, and secretion of large amounts of autoantibodies (e.g., anti-dsDNA antibodies, anti-Sm antibodies). These autoantibodies bind to self-antigens to form immune complexes, which deposit in tissues and organs, triggering inflammatory injury (e.g., lupus nephritis, skin erythema) [1]. Under the pathological state of SLE, the heterogeneity, clonal evolution, CDR3 repertoire characteristics, and the immunopathological and regulatory effects exerted by self-reactive B cell subsets have not yet been fully elucidated [2,3].

According to the classical clonal selection theory, during B cell development and differentiation, mechanisms such as VDJ allelic exclusion and stringent self-tolerance selection ensure that B cells exported to the periphery express only one type of B cell receptor (BCR) [4]. However, incomplete VDJ allelic exclusion (due to allelic exclusion escape or allelic inclusion) results in the presence of a certain proportion of dual BCR B cells in the peripheral immune system, which have been shown to be closely associated with B cell development, senescence, and autoimmune responses [5,6,7,8,9,10]. In 2015, Fraser et al. found that some SLE patients exhibited a high enrichment of B cells co-expressing Igκ and Igλ, and proposed that dual BCR-expressing cells may enhance the potential reactivity of B cells to antigens [11]. In 2018, Sang et al. found that the proportion of dual BCR B cells was elevated in SLE patients and MRL/Lpr mice. These cells exhibited stronger responses to Toll-like receptor 7/9 and type I/II interferon stimulation, higher levels of MHC-II and co-receptor molecules, and depended on IL-21 for homeostasis maintenance, displaying self-reactive characteristics [12]. In 2022, Peterson et al. found that in a subset of adult SLE patients, dual BCR B cells highly expressed *VH4-34* and were abnormally activated, exhibiting enhanced responses to TLR7 and TLR9 signaling, which were further driven by an enhanced type II IFN response [13]. However, the BCR pairing types, proportion, CDR3 repertoire characteristics, subset origin distribution, clonal evolution patterns, and corresponding effector molecule expression of dual BCR B cells in SLE have not yet been elucidated.

The combination of scRNA-seq and scBCR-seq technologies provides a powerful tool for dissecting the transcriptomic expression and BCR clonal evolution characteristics of different B cell subsets at the high-throughput single-B-cell level [14,15,16]. Leveraging the breakthrough advances of scRNA-seq combined with scBCR-seq technology, this study used MRL/Lpr model mice, SLE.Yaa model mice, and SLE patients as research subjects to, for the first time, dissect the relationship between dual BCR B cells and SLE at the high-throughput single-B-cell level, thereby providing fundamental data and novel perspectives for in-depth investigation of B cell response effects and mechanisms in SLE.

## 2. Materials and Methods

### 2.1. Research Subjects and Samples

MRL/Lpr mice: MRL/Lpr mice (*n* = 3), female, 12 weeks old, purchased from Huachuang Xinnuo Pharmaceutical Technology Co., Ltd., Taizhou, China. Healthy C57BL/6 mice (*n* = 3), female, 12 weeks old, purchased from the Laboratory Animal Center of Zunyi Medical University. The mice were housed in separate cages in an SPF facility. On day 5 of the 13th week, spleen samples were collected from each mouse for single-cell sequencing. MRL/Lpr mice are referred to as the MRL group; healthy C57BL/6 mice are referred to as the H group.

SLE.Yaa mice: Shared scRNA + BCR-seq data of spleen samples from SLE.Yaa (male, 15 weeks old) mice and control mice (C57BL/6, male, 15 weeks old) were obtained from a study published in the Journal of Immunology on 27 April 2022, under the title “Lupus susceptibility loci predispose mice to clonal lymphocytic responses and myeloid expansion.” The shared data are deposited in the public database Gene Expression Omnibus (GEO) under accession number GSE192762. SLE.Yaa (B6) mice are referred to as Group B (*n* = 2); healthy C57BL/6 mice are referred to as Group C (*n* = 2). Both SLE.Yaa and control mice in the study were purchased from The Jackson Laboratory and housed in an AAALAC-accredited facility at Harvard Medical School.

SLE patients and healthy controls (all female): scRNA + BCR-seq data of peripheral blood were obtained from a study published in Science Immunology on 18 March 2022, under the title “Two subsets of human marginal zone B cells resolved by global analysis of lymphoid tissues and blood.” The shared data are deposited in the public database Gene Expression Omnibus (GEO) under accession number GSE193867. SLE patients are referred to as the SLE group (*n* = 3); healthy control volunteers are referred to as the HC group (*n* = 3). (Basic information and ethical approval for SLE patients and healthy controls were obtained from the original study; a brief description is provided in Supplementary Figure S9A).

### 2.2. Preparation of Single-Cell Suspensions from Mouse Tissue Samples

MRL/Lpr mice and healthy C57BL/6 mice: Under sterile conditions, fresh mouse spleen tissues were collected using a scalpel. Tissues were washed twice with pre-chilled RPMI 1640 medium containing 0.04% BSA. The tissues were thoroughly minced with surgical scissors into small pieces of approximately 0.5 mm^3^ and placed into freshly prepared enzyme digestion solution, followed by enzymatic digestion in a 37 °C constant-temperature incubator for 30–60 min, with mixing by inversion every 5–10 min. The digested cell suspension was filtered once or twice through a BD 40 μm cell strainer and centrifuged at 300× *g* for 5 min at 4 °C. The pellet was resuspended in an appropriate volume of medium, and an equal volume of red blood cell lysis buffer (MACS, Cat. No. 130-094-183) (Miltenyi Biotec, Bergisch Gladbach, Germany) was added. After mixing, the suspension was incubated at 4 °C for 10 min, then centrifuged at 300× *g* for 5 min, and the supernatant was discarded. The pellet was washed once with medium, centrifuged at 300× *g* for 5 min, and the supernatant was discarded. The cell pellet was resuspended in 100 μL of medium, and cell concentration and viability were determined using a Luna cell counter(Thermo Fisher Scientific, Wuhan, China). Dead cell removal was performed according to the instructions of the MACS Dead Cell Removal Kit (130-090-101) (Miltenyi Biotec, Bergisch Gladbach, Germany). 100 μL of magnetic beads were added to the cell suspension, mixed by pipetting, incubated at room temperature for 15 min, then passed through a column. The eluted cell suspension was centrifuged at 300× *g* for 5 min at 4 °C, washed twice, resuspended in 50–100 μL of medium, and counted using a Luna counter. From the prepared single-cell suspension, CD45-positive cells were isolated from 10^7^ splenocytes per sample using CD45 MicroBeads (Miltenyi Biotec, Cologne, Germany). The sorted cells were counted with a Luna counter and then used for scRNA-seq.

### 2.3. Flow Cytometry and Immunohistochemistry

Spleens were harvested from 6 healthy C57BL/6 mice (13 weeks old, female) and 4 MRL/Lpr model mice (13 weeks old, female) for flow cytometry validation. The antibodies were purchased from BioLegend Inc. (San Diego, California, United States), with the catalog numbers 409509 (anti-mouse Ig light chain κ, FITC) and 407307 (anti-mouse Ig light chain λ, PE), respectively. Spleens, kidneys, livers and lymph nodes were collected from one MRL/Lpr model mouse (13 weeks old, female) and an age-matched healthy C57BL/6 mouse (13 weeks old, female) for immunohistochemical assays. The antibody was purchased from BioLegend Inc. with the catalog number 110501 (Purified anti-mouse CD19 Antibody). MRL/Lpr mice were purchased from Jiangsu Huachuang Xinnuo Pharmaceutical Technology Co., Ltd., Taizhou, China. Healthy C57BL/6 mice were obtained from the Experimental Animal Center of Zunyi Medical University. The mice used for flow cytometry and immunohistochemical assays were from the same batch as those subjected to single-cell sequencing.

### 2.4. Construction and Sequencing of 5′ Transcriptome Libraries

The freshly prepared single-cell suspension was adjusted to a concentration of 700–1200 cells/μL. According to the 10× Genomics Chromium Next GEM Single Cell 5′ Reagent Kits v2.0 (Cat. No. 1000165) (10× Genomics, Pleasanton, California, United States) user manual, droplet generation and cDNA library amplification were performed. Library construction was carried out using the Chromium™ Single Cell 3′/5′ Library Construction Kit (Cat. No. 1000020). The constructed libraries were sequenced (Shanghai OE Biotech Co., Ltd., Shanghai, China). The raw data generated by high-throughput sequencing were in FASTQ format. The official 10× Genomics software Cell Ranger (v5.0.0) was used for data quality statistics and alignment to the reference genome. This software quantifies high-throughput single-cell transcriptome data by identifying cell-specific barcode sequences and UMI (Unique Molecular Identifier) sequences for each mRNA molecule within each cell. Sequencing data, average reads per cell, UMI Q30 values, etc. (Supplementary Figure S1C) were obtained to ensure that the data met the criteria for analysis.

### 2.5. Single-Cell 5′V(D)J Data Processing

For single-cell immune repertoire sequencing data, the cellranger vdj command from the official 10× Genomics software Cell Ranger (version 7.0.1) was used with the parameter: refdata-cellranger-vdj-GRCm38-alts-ensembl-7.0.0 to perform V(D)J sequence assembly and paired clone type identification for each sample. Cells were typed based on their CDR3 sequences. Cells with identical CDR3 cDNA sequences for the BCR were assigned to the same clone type. Contigs belonging to cells of the same clone type were further mixed and assembled, and polymorphic sites were integrated to generate a consensus sequence for that clone type. The Seurat package (version 4.0.0) was used to integrate clone type information for each cell into the single-cell gene expression analysis. Downstream analyses were performed using VDJtools (version 1.1.4).

### 2.6. Analytical Workflow for Single BCR B Cells and Dual BCR B Cells

**Figure 1:**
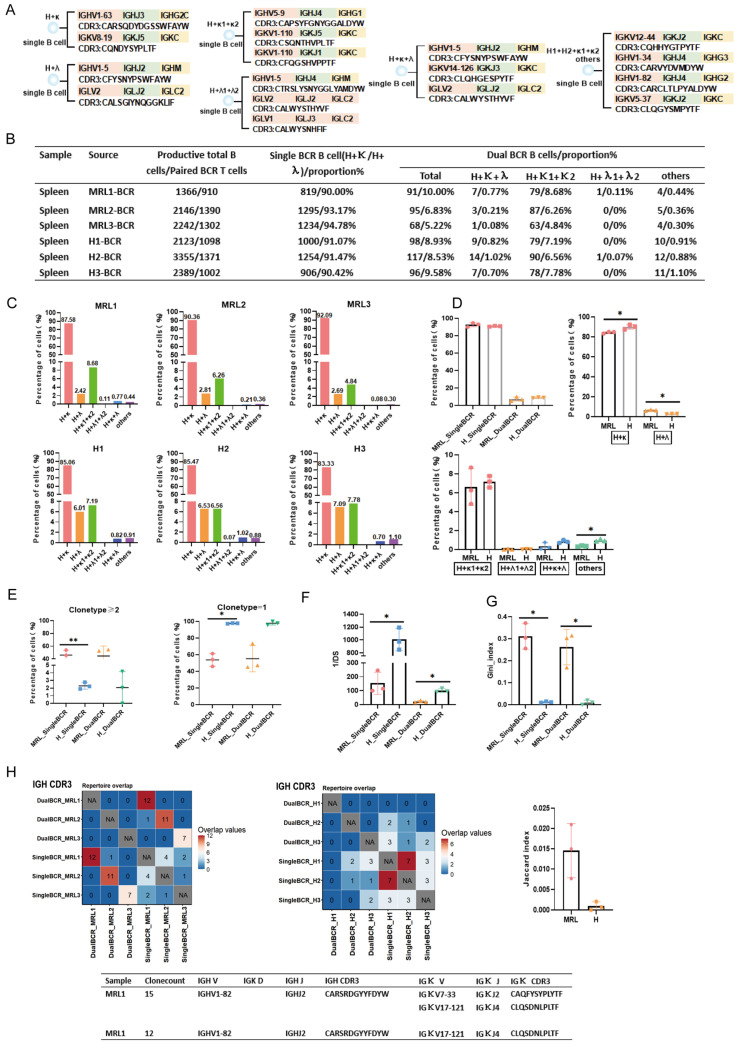
scBCR-seq analysis of single BCR B cells and dual BCR B cells in the spleen of MRL/Lpr mice and healthy C57BL/6 mice. (**A**) Examples of CDR3 sequences from individual B cells showing BCR heavy chain (H), kappa (κ), and lambda (λ) pairing types (H + κ; H + λ; H + κ1 + κ2; H + λ1 + λ2; H + κ + λ; others). (**B**) Sample names; scRNA-seq accession numbers; total number of functional B cells; scBCR-seq accession numbers; total number of paired BCR B cells; BCR pairing types and proportions. (**C**) BCR pairing types and proportions for each mouse. (**D**) Statistical comparison of BCR pairing type proportions between the MRL group and the H group. (**E**) Statistical comparison of clonal expansion (clone type ≥ 2; clone type = 1) proportions for single BCR and dual BCR B cells between the MRL group and the H group. (**F**) Statistical comparison of diversity (1/DS) of single BCR and dual BCR B cells between the MRL group and the H group. (**G**) Statistical comparison of the Gini index of single BCR B cells and dual BCR B cells between the MRL group and the H group. (**H**) Overlap analysis of IGH CDR3 sequences of single and dual BCR B cells in mice from the MRL group (**left panel**) and the H group (**right panel**); comparison of Jaccard overlap index; examples of shared clonal sequences between single BCR B cells and dual BCR B cells. (* *p* < 0.05, ** *p* < 0.01).

scBCR-seq data analysis workflow: (1) Remove sequences marked as “FALSE” in the “is_cell” and “high_confidence” columns; exclude sequences with chains other than “IGH”, “IGK”, or “IGL” in the “Chain” column; remove sequences marked as “Non” or “FALSE” in the “Productive” column; discard B cells for which only a single functional chain was sequenced. (2) In a single B cell, two functional chains that can pair and assemble are defined as a single BCR B cell, specifically BCR heavy chain (H) + BCR kappa or BCR lambda chain (H + κ; H + λ). In a single B cell, three or more functional chains that can pair and assemble are uniformly defined as a dual BCR B cell. These mainly include: (1) H + κ1 + κ2; (2) H + λ1 + λ2; (3) H + κ + λ; (4) others: H1 + H2 + κ; H1 + H2 + λ; H1 + H2 + κ + λ, etc. For each BCR pairing type, one B cell was selected, and the V(D)J gene family name and the CDR3 amino acid sequence are listed (Figure 1A, Figure 3A and Figure 5A).

### 2.7. scBCR-Seq Analysis of CDR3 Repertoire Characteristics

After quality control of the scBCR-seq data, the immunarch package in R was used to compare and analyze the CDR3 repertoire characteristics of single BCR B cells and dual BCR B cells: (1) Clonality: Comparative analysis of the proportion of cells with clonal count ≥ 2 and the proportion of cells with clonal count = 1 in single BCR B cells versus dual BCR B cells. Diversity was calculated using the inverse Simpson index (1/DS): 1/DS = (∑ni)^2^/∑(ni^2^). Diversity was compared between single BCR B and dual BCR B cells across groups. For within-group individual analysis, the composition and characteristics of B cell numbers derived from single-cell sequencing were analyzed; for between-group statistical comparisons, data were based on total values from individual biological subjects (statistical comparisons were not performed for data not meeting statistical criteria). (2) V gene usage preference: Comparative analysis of V gene subfamily usage between single BCR B cells and dual BCR B cells was performed using SPSS (version 26.0) to compare differences in V gene usage. (3) Overlap: Overlap of CDR3 sequences was analyzed using the immunarch package (version 0.10.3) in R. (4) Gini index: The immune repertoire data pre-processed by MiXCR software (version 4.3.2) were loaded using the immunarch R package. Clonal amino acid sequences (CDR3.aa) and their absolute frequencies (clones) were extracted from each sample, and clones with zero frequency or invalid entries were filtered out. The Gini index (G) was calculated using the following formula:

G = Σ|p_i − p_j|/(2 × n^2^ × μ),

where p_i and p_j represent the frequencies of any two clones, n is the total number of clones, and μ is the mean frequency of all clones. This calculation was implemented via a custom function. (5) Integration of scRNA-seq and scBCR-seq data for “effector molecule expression and receptor clone type” tracking analysis using the unique barcode of each B cell.

### 2.8. scRNA-Seq Data Quality Control and Integrated Tracking Analysis with scBCR-Seq Data

The scRNA-seq data sequenced in this experiment and the scRNA-seq data shared in the GEO database were imported into the R environment for analysis. (1) Data quality control: Cells with detected gene counts < 200 or >3000, total read counts < 1000, or mitochondrial RNA content > 5% were removed. (2) Doublet detection and removal were performed using the R package DoubletFinder (version 2.0) in combination with Seurat V5 (Note: the total number of doublet cells removed by DoubletFinder in this experiment is shown in Supplementary Figure S1D). (3) Data normalization was performed using the LogNormalize method in the Seurat package (V4.0.1) with a scale factor of 10,000. (4) The functions ScaleData, FindVariableFeatures, and RunPCA were subsequently used for data scaling, highly variable gene selection, and principal component analysis. Non-linear dimensionality reduction and cell clustering were performed using the RunUMAP function. The FindAllMarkers function was used to identify the top 10 differentially expressed mRNAs for each cell subpopulation. Cell type annotation was performed using the SingleR package (version 1.4.1), and clustering results were refined based on the expression levels of canonical marker genes. (5) Re-clustering analysis of B cell subpopulations was performed, and subpopulations were defined according to the expression of canonical marker genes: Naive_B (*Cd19*, *Ighd*); Memory_B (*Cd19*, *Cd40*, *Tnfrsf13b*, *Aim2*); Breg (*Cd19*, *Il10*); Plasma (*Cd19*, *Jchain*, *Xbp1*), (6) B cells that met both the scRNA-seq quality control criteria and the scBCR-seq filtering conditions were subjected to tracking analysis based on their unique cellular barcodes. (7) Gene set enrichment analysis (GSEA): The msigdbr package was used to obtain the Hallmark gene sets for human and mouse as annotation backgrounds. Fast GSEA was performed using the fgsea package with parameters set to minSize = 15, maxSize = 500, and nperm = 10,000. (8) Differential gene expression analysis: Differentially expressed genes between groups were identified using the FindMarkers function in Seurat with the Wilcoxon rank-sum test, setting logfc.threshold = 0.25 and min.pct = 0.1. After correction by the Benjamini–Hochberg method, an adjusted *p*-value (p_val_adj) < 0.05 and |log_2_FC| > 0.5 were used as criteria to define differentially expressed genes. The results were visualized using volcano plots, with the top 10 upregulated and downregulated genes annotated.

### 2.9. Main Software Tools and Statistical Methods

Software packages: The R packages Seurat (version 4.0.0), ggplot2 (version 4.0), and DoubletFinder (version 2.0), as well as Adobe and GraphPad Prism (v5), were used for graphing. Data analysis was performed using R Studio (v4.3.2) and GraphPad Prism (v5). Cell types were annotated using the SingleR software package (version 1.4.1), and the annotation results were manually corrected based on the expression of canonical marker genes. Statistical methods: Statistical analysis was performed using IBM SPSS Statistics 26 software. When defining cells by n, for n ≥ 40 and Tmin ≥ 5, the Pearson chi-square test was used to compare multiple ratios between two groups; for n < 40 or Tmin < 1, Fisher’s exact test was used. For comparisons of B cell clone types between two groups, the paired *t*-test was used; if the data were not normally distributed, a non-parametric test was applied. The Gini index and diversity were analyzed using one-way ANOVA. For statistical analysis of differential gene expression, the Wilcoxon rank-sum test was used. * *p* < 0.05, ** *p* < 0.01, *** *p* < 0.001, **** *p* < 0.0001.

## 3. Result

### 3.1. Dual BCR B Cells in the Spleen of MRL/Lpr Mice

Spleen tissues were collected from 3 female MRL/Lpr mice and 3 female healthy C57BL/6 mice. MRL/Lpr mice exhibited splenomegaly-like features (Supplementary Figure S1B). Immunohistochemistry (CD19) was performed on the spleen, kidney, liver, and lymph node tissues of MRL/Lpr mice and healthy C57BL/6 mice. The proportion of B cells in MRL/Lpr mice was increased compared with that in healthy C57BL/6 mice (Supplementary Figure S1A).

Flow cytometry (FCM) results of spleen tissues from MRL/Lpr (S1, S2, S3, S4) and healthy C57BL/6 mice (H1, H2, H3, H4, H5, H6) showed that approximately 1.29–4.54% of cells in the MRL group co-expressed kappa and lambda chains; similarly, approximately 1.55–5.37% of cells in the H group co-expressed kappa and lambda chains (Supplementary Figure S2B).

Proportion of dual BCR B cells and CDR3 repertoire characteristics: In the MRL group, approximately 7.05% of B cells were dual BCR B cells. A schematic representation of the sequences is shown in Figure 1A. The main pairing type was H + κ1 + κ2 (8.68%, 6.26%, 4.24%), followed by the “others” type mainly consisting of H1 + H2 + κ (0.44%, 0.36%, 0.30%) and H + κ + λ (0.77%, 0.21%, 0.08%). In the H group, approximately 8.96% of B cells were dual BCR B cells, with the main pairing type also being H + κ1 + κ2 (7.19%, 6.60%, 7.82%), followed by the “others” type mainly consisting of H1 + H2 + κ1 + κ2 (0.91%, 0.88%, 1.10%) and H + κ + λ (0.82%, 1.02%, 0.70%). Differences in the proportion of the “others” pairing type (mainly H1 + H2 + κ + λ) of dual BCR B cells were observed between the MRL and H groups (Figure 1B–D). The proportion of single BCR B cells with clone type count ≥ 2 was significantly higher in the MRL group than in the H group, and the proportion of dual BCR B cells with clone type count ≥ 2 was slightly higher in the MRL group than in the H group, whereas the diversity of dual BCR B cells was lower in the MRL group than in the H group (Figure 1E,F). The Gini index of both single and dual BCR B cells was higher in the MRL group than in the H group (Figure 1G). No shared CDR3 sequences were found between single BCR B cells and dual BCR B cells in either the MRL or H groups. However, within the MRL group, partial sharing of CDR3 sequences existed between single BCR B cells and dual BCR B cells, and the Jaccard overlap index was higher in the MRL group than in the H group; some of the partially shared CDR3 sequences originated from clonally expanded B cells (Figure 1H). In the MRL group, *IGHV1*, *IGHV9*, and *IGHJ2* usage was significantly higher in dual BCR B cells than in single BCR B cells. In the H group, *IGHV11* (and other *IGHV11* family members) usage was significantly higher in dual BCR B cells than in single BCR B cells. *IGHV1* and *IGHJ4* usage in dual BCR B cells was significantly higher in the MRL group than in the H group (Supplementary Figure S3).

**Figure 2:**
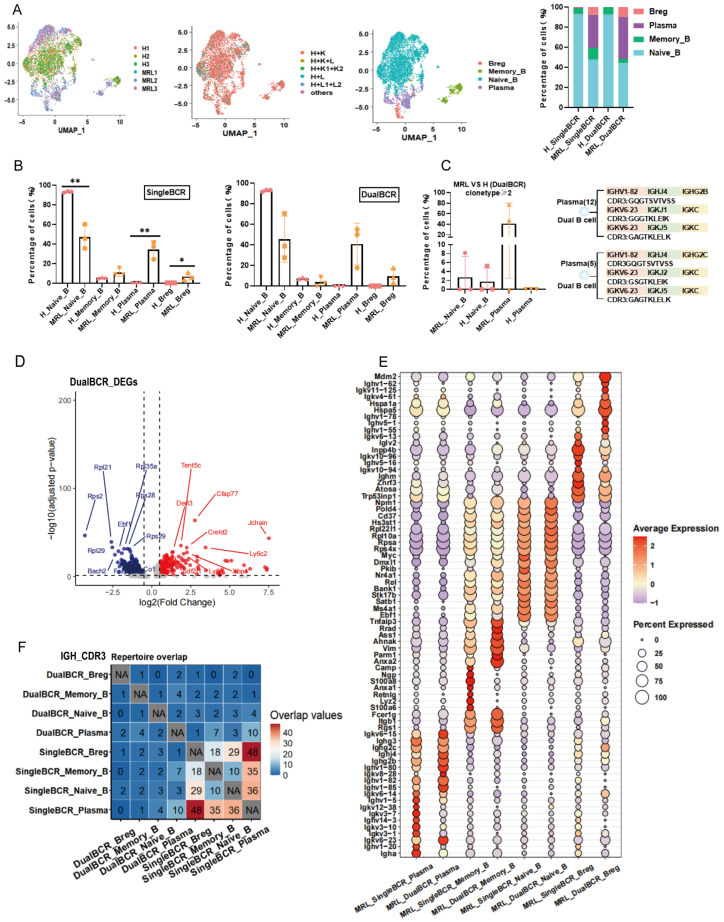
scRNA + BCR-seq analysis of the subset origins and characteristics of single BCR B cells and dual BCR B cells in the spleen of MRL/Lpr mice and healthy C57BL/6 mice. (**A**) Proportions and distributions of four B cell subpopulations in the MRL group and the H group; proportional distribution of the four B cell subpopulations; proportions and distributions of TCR pairing types within the four B cell subpopulations; stacked bar graphs of single BCR B cells and dual BCR B cells within the four B cell subpopulations in the MRL group and the H group. (**B**) Proportions of single BCR B cells and dual BCR B cells within the four B cell subpopulations in the MRL group and the H group. (**C**) Clonal expansion proportions (clone type ≥ 2) of dual BCR B cell subpopulations in the MRL group and the H group; display of sequences from clonally expanded cells. (**D**) Volcano plot of differentially expressed genes in dual BCR B cells comparing the MRL group with the H group. (**E**) Comparison of the top 10 mRNA expressions of single and dual BCR B cell subpopulations in the MRL group. (**F**) Number of overlapping IGH CDR3 sequences among the four single and dual BCR B cell subpopulations in the MRL group. (* *p* < 0.05, ** *p* < 0.01).

Subpopulation distribution of single BCR B cells: MRL group: Naive_B (47.24% ± 12.52%) > Plasma (34.65% ± 8.83%) > Memory_B (11.27% ± 3.98%) > Breg (6.84% ± 3.65%). H group: Naive_B (93.27% ± 0.64%) > Memory_B (5.62% ± 0.59%) > Plasma (0.68% ± 0.12%) > Breg (0.43% ± 0.08%). Subpopulation distribution of dual BCR B cells: MRL group: Naive_B (45.66% ± 22.38%) > Plasma (40.88% ± 19.20%) > Breg (9.59% ± 7.30%) > Memory_B (3.87% ± 4.42%). H group: Naive_B (92.74% ± 1.00%) > Memory_B (7.26% ± 1.03%). The proportion of single BCR Naive_B subpopulation in the MRL group was significantly lower than that in the H group, whereas the proportions of single BCR Plasma and Breg subpopulations were higher in the MRL group than in the H group. The proportions of dual BCR Plasma and Breg subpopulations were both higher in the MRL group than in the H group (Figure 2A,B). In the MRL group, the proportion of dual BCR B cells with clone type count ≥ 2 was: Plasma (47.13% ± 38.70%) > Naive_B (2.72% ± 4.71%) (Figure 2C). In the H group, the proportion of dual BCR B cells with clone type count ≥ 2 was: Naive_B (1.75% ± 3.04%) (Figure 2C). Clonal expansion of dual BCR Plasma and Naive_B cells was higher in the MRL group than in the H group. The CDR3 sequences of clonally expanded dual BCR Plasma cells are shown in Figure 2C. For single BCR B cell subpopulations with clone type count ≥ 2: MRL_singleBCR_Naive_B (10.02% ± 4.51%) > H_singleBCR_Naive_B (1.22% ± 0.36%); MRL_singleBCR_Memory_B (58.20% ± 13.0%) > H_singleBCR_Memory_B (0.64% ± 1.39%). In the MRL group, single BCR Plasma and Breg cells exhibited clone type count ≥ 2 at 56.91% ± 10.54% and 18.62% ± 16.21%, respectively, whereas no cells with clone type count ≥ 2 were found for single BCR Plasma or Breg cells in the H group (Supplementary Figure S4A,B,D).

Differential gene expression: In the MRL group, dual BCR B cells up-regulated genes such as Ly6a, Ly6c2, *Jchain*, and *Xbp1*, and down-regulated genes such as *Rps2*, *Rpl21*, *Rpl35a*, and *Bach2* (Figure 2D). The up-regulated and down-regulated genes in single BCR B cells of the MRL group were partially consistent with those in dual BCR B cells (Supplementary Figure S5A). Expression of *Ifitm1*, *Ifitm2*, *Isg15*, *Bst2*, and *Mx1* in single BCR B cells was significantly higher in the MRL group than in the H group (Supplementary Figure S5C). Expression of *Isg15*, *Isg20*, *Bst2*, and *Mx1* in dual BCR B cells was significantly higher in the MRL group than in the H group (Supplementary Figure S5E). GSEA: In the MRL group, single BCR B cells significantly enriched pathways such as GLYCOLYSIS and UV_RESPONSE_UP, whereas the MITOTIC_SPINDLE pathway was down-regulated (Supplementary Figure S5D). For dual BCR B cells, the MRL group enriched pathways including GLYCOLYSIS and UNFOLDED_PROTEIN_RESPONSE (NES > 1, *p* < 0.05), while the ADHERENS_JUNCTION pathway was down-regulated (Supplementary Figure S5F). The composition of enriched pathways differed between dual BCR B cells and single BCR B cells. The top 10 mRNAs were largely consistent among each single BCR B and dual BCR B cell subpopulation within both the MRL and H groups (Figure 2E and Figure S4C). In the MRL group, shared CDR3 sequences existed among the four single BCR B and dual BCR B cell subpopulations, but the sharing among single BCR B cell subpopulations was higher (Figure 2F). In the H group, shared CDR3 sequences among the four single BCR B and dual BCR B cell subpopulations were markedly lower than those in the MRL group (Supplementary Figure S4E).

### 3.2. Dual BCR B Cells in the Spleen of SLE.Yaa Mice

**Figure 3:**
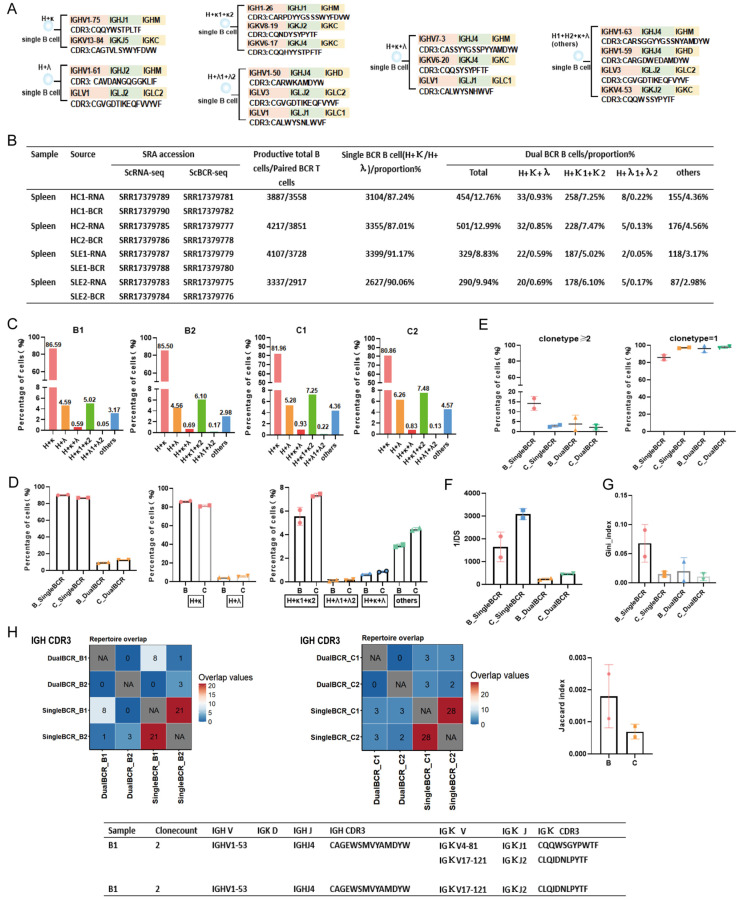
scBCR-seq analysis of CDR3 repertoire characteristics of single BCR B cells and dual BCR B cells in the spleen of SLE model mice (referred to as Group B) and healthy control mice (referred to as Group C). (**A**) Examples of CDR3 sequences from individual B cells showing different BCR heavy chain (H), kappa (κ), and lambda (λ) pairing types (H + κ; H + λ; H + κ1 + κ2; H + λ1 + λ2; H + κ + λ; others). (**B**) Sample names; scRNA-seq accession numbers; total number of functional B cells; scBCR-seq accession numbers; total number of paired BCR B cells; BCR pairing types and proportions. (**C**) BCR pairing types and proportions for individual samples. (**D**) Overall BCR pairing types and proportions; proportions of total single BCR B cells and total dual BCR B cells. (**E**) Analysis of clonal expansion (clone type ≥ 2) proportions of single BCR and dual BCR B cells in Group B and Group C mice. (**F**) Comparison of diversity (1/DS) of single BCR and dual BCR B cells in Group B; comparison of diversity (1/DS) of single BCR and dual BCR B cells in Group C; comparison of diversity (1/DS) of dual BCR B cells between Group B and Group C. (**G**) Gini index analysis of single BCR B cells and dual BCR B cells in Group B and Group C mice. (**H**) Overlap analysis of IGH CDR3 sequences of single and dual BCR B cells in Group B (**left panel**) and Group C (**right panel**); comparison of Jaccard overlap index; examples of shared clonal sequences between single BCR B cells and dual BCR B cells.

Proportion of dual BCR B cells and CDR3 repertoire characteristics: In Group B, approximately 9.32% of B cells were dual BCR B cells. A schematic representation of the sequences is shown in Figure 3A. The main pairing type was H + κ1 + κ2 (5.02%, 6.10%), followed by the “others” type mainly consisting of H1 + H2 + κ1 + κ2 (3.17%, 2.98%) and H + κ + λ (0.59%, 0.69%). In Group C, approximately 12.88% of B cells were dual BCR B cells, with the main pairing type also being H + κ1 + κ2 (7.25%, 7.48%), followed by the “others” type mainly consisting of H1 + H2 + κ1 + κ2 (4.36%, 4.56%) and H + κ + λ (0.93%, 0.83%) (Figure 3B–D). The clonal expansion (clone type count ≥ 2) of both single BCR B cells and dual BCR B cells was higher in Group B than in Group C, whereas the diversity of dual BCR B cells was lower in Group B than in Group C (Figure 3E,F). The Gini index of both single BCR B cells and dual BCR B cells was higher in Group B than in Group C (Figure 3G). No overlapping CDR3 sequences were found in dual BCR B cells between Group B and Group C. Compared with Group C, Group B had more overlapping CDR3 sequences in single BCR B cells; some of the partially shared CDR3 sequences originated from clonally expanded B cells, and the Jaccard overlap index was higher in Group B than in Group C (Figure 3H). Dual BCR B cells in Group B preferentially used *IGHV8* and *IGHJ4* genes, whereas those in Group C preferentially used *IGHV5*, *IGHV10*, and *IGHJ2* genes (Supplementary Figure S6).

**Figure 4:**
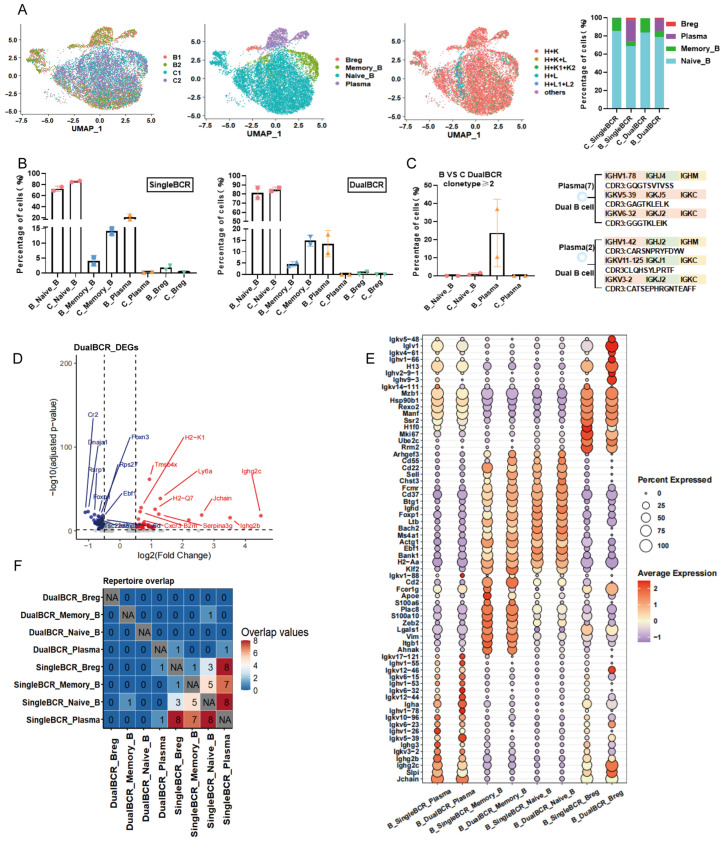
scRNA + BCR-seq analysis of the subset origins and characteristics of single BCR B cells and dual BCR B cells in the spleen of SLE model mice (referred to as Group B). (**A**) Proportions and distributions of four B cell subpopulations in the SLE group and the healthy group; proportional distribution of the four B cell subpopulations; proportions and distributions of TCR pairing types within the four B cell subpopulations; stacked bar graphs of single BCR B cells and dual BCR B cells within the four B cell subpopulations in Group B and Group C. (**B**) Proportional analysis of single BCR B cells and dual BCR B cells within the four B cell subpopulations in Group B and Group C. (**C**) Expansion proportions of dual BCR B cell subpopulations with clone type count ≥ 2 in Group B and Group C; display of sequences from clonally expanded cells. (**D**) Volcano plot of differentially expressed genes in dual BCR B cells comparing Group B with Group C. (**E**) Comparison of the top 10 mRNA expressions of single and dual BCR B cell subpopulations in Group B. (**F**) Number of overlapping IGH CDR3 sequences among the four single and dual BCR B cell subpopulations in Group B.

Subpopulation distribution of single BCR B cells: Group B: Naive_B (72.55%) > Plasma (21.50%) > Memory_B (4.14%) > Breg (1.82%). Group C: Naive_B (85.80%) > Memory_B (14.11%) > Plasma (0.32%) > Breg (0.11%). Subpopulation distribution of dual BCR B cells: Group B: Naive_B (81.37%) > Plasma (13.50%) > Memory_B (4.56%) > Breg (0.57%). Group C: Naive_B (85.16%) > Memory_B (14.84%). The proportion of the single BCR Naive_B subpopulation in Group B was significantly lower than that in Group C, whereas the proportions of single BCR Plasma and Breg subpopulations were higher in Group B than in Group C. The proportions of dual BCR Plasma and Breg were both higher in Group B than in Group C (Figure 4A,B). In Group B, only Plasma cells within the dual BCR B cell subpopulation exhibited clonal expansion (clone type count ≥ 2), with a proportion of 29.23%. In Group C, only Naive_B cells within the dual BCR B cell subpopulation exhibited clonal expansion (clone type count ≥ 2), with a proportion of 0.88% (Figure 4C). For clonal expansion in single BCR B cell subpopulations in Groups B and C: B_singleBCR_Naive_B (2.18%) > C_singleBCR_Naive_B (1.40%); B_singleBCR_Memory_B (5.34%) > C_singleBCR_Memory_B (3.60%). In Group B, single BCR Plasma and Breg cells exhibited clonal expansion at 40.10% and 11.06%, respectively, whereas no clonal expansion was observed for single BCR Plasma or Breg cells in Group C (Supplementary Figure S7A,B,D).

Differential gene expression: In Group B, dual BCR B cells up-regulated genes such as *Ly6a*, *Ighg2b*, and *Jchain*, and down-regulated genes such as *Rps27*, *Rsrp1*, and *Ebf1* (Figure 4D). The up-regulated and down-regulated genes in single BCR B cells of Group B were partially consistent with those in dual BCR B cells (Supplementary Figure S8A). The top 10 mRNA expressions of single BCR B and dual BCR B cells in Groups B and C were consistent (Figure 4E and Figure S7C). Expression of *Ifitm1*, *Ifitm2*, *Isg20*, *Isg15*, and *Ifi27* in single BCR B cells was significantly higher in Group B than in Group C (Supplementary Figure S8C). Expression of *Ifitm1*, *Isg20*, *Irf7*, and *Ifi27* in dual BCR B cells was significantly higher in Group B than in Group C (Supplementary Figure S8E). GSEA: In Group B, single BCR B cells significantly enriched pathways such as GLYCOLYSIS and UNFOLDED_PROTEIN_RESPONSE, whereas MITOTIC_SPINDLE and MYOGENESIS pathways were down-regulated (Supplementary Figure S8D). Compared with Group C, dual BCR B cells in Group B specifically enriched interferon signaling pathways, including INTERFERON_GAMMA_RESPONSE and INTERFERON_ALPHA_RESPONSE, as well as metabolic pathways such as GLYCOLYSIS. Down-regulated pathways included MITOTIC_SPINDLE (Supplementary Figure S8F). Compared with single BCR B cells, activation of the interferon-gamma signaling pathway was a unique characteristic of dual BCR B cells. No CDR3 overlap was found between dual BCR B cell subpopulations in Groups B and C (Figure 4F and Figure S7E). Partial CDR3 overlap existed between single BCR B and dual BCR B cell subpopulations in both Groups B and C, and the number of overlapping CDR3 sequences was higher in Group B (Figure 4F and Figure S7E).

### 3.3. Dual BCR B Cells in the Peripheral Blood of SLE Patients

**Figure 5:**
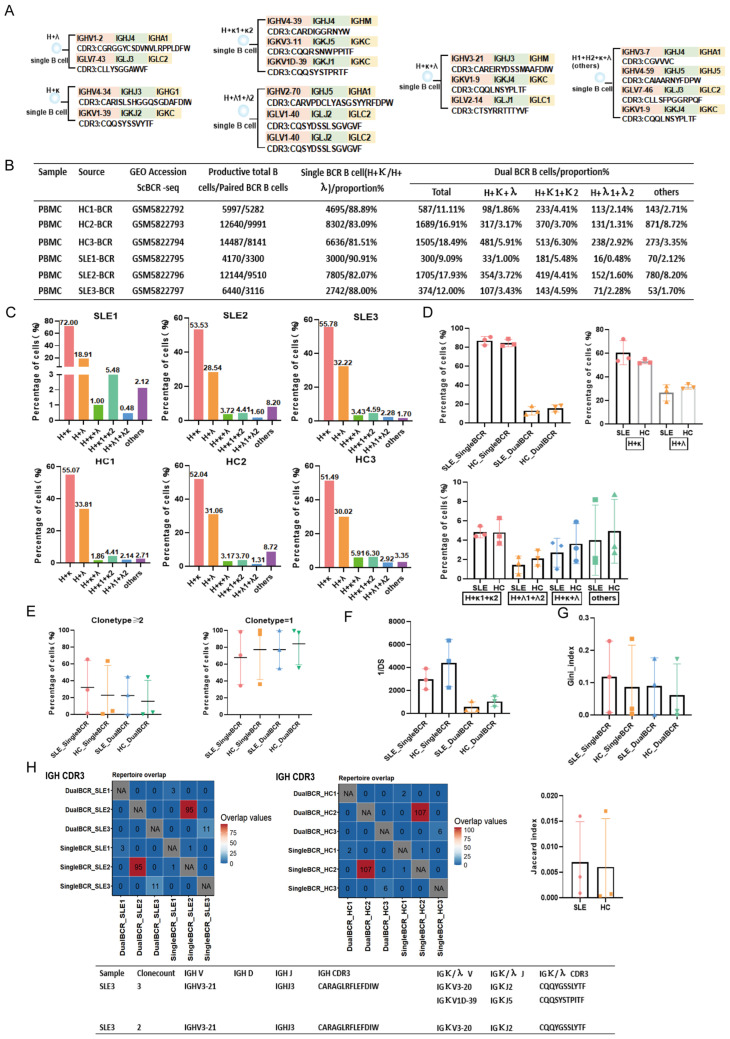
scBCR-seq analysis of CDR3 repertoire characteristics of single BCR B cells and dual BCR B cells in peripheral blood of SLE patients and healthy control volunteers. (**A**) Examples of CDR3 sequences from individual B cells showing different BCR heavy chain (H), kappa (κ), and lambda (λ) pairing types (H + κ; H + λ; H + κ1 + κ2; H + λ1 + λ2; H + κ + λ; others). (**B**) Sample names; scRNA-seq accession numbers; total number of functional B cells; scBCR-seq accession numbers; total number of paired BCR B cells; BCR pairing types and proportions. (**C**) BCR pairing types and proportions for each patient and healthy control. (**D**) Overall BCR pairing types and proportions; proportions of total single BCR B cells and total dual BCR B cells. (**E**) Analysis of clonal expansion (clone type ≥ 2) proportions of single BCR and dual BCR B cells in SLE patients and healthy controls. (**F**) Comparison of diversity (1/DS) of single BCR and dual BCR B cells in SLE patients; comparison of diversity (1/DS) of single BCR and dual BCR B cells in healthy controls; comparison of diversity (1/DS) of dual BCR B cells between SLE patients and healthy controls. (**G**) Gini index analysis of single BCR B cells and dual BCR B cells in the SLE group and the HC group. (**H**) Overlap analysis of IGH CDR3 sequences of single and dual BCR B cells in the SLE group (**left panel**) and the HC group (**right panel**); comparison of Jaccard overlap index; examples of shared clonal sequences between single BCR B cells and dual BCR B cells.

Proportion of dual BCR B cells and CDR3 repertoire characteristics: In the SLE group, approximately 14.94% of B cells were dual BCR B cells. A schematic representation of the sequences is shown in Figure 5A. The dual BCR B cells were mainly of the “others” type dominated by H1 + H2 + κ1 + κ2 (2.12%, 8.20%, 1.70%), followed by the H + κ1 + κ2 type (5.48%, 4.41%, 4.59%) and the H + κ + λ type (1.00%, 3.72%, 3.43%). In the HC group, approximately 16.15% of B cells were dual BCR B cells, with the main type being H + κ1 + κ2 (4.41%, 3.70%, 6.30%), followed by the “others” type dominated by H1 + H2 + κ1 + κ2 (2.71%, 8.72%, 3.35%) and the H + κ + λ type (1.86%, 3.17%, 5.91%) (Figure 5B–D). The proportion of cells with clone type count ≥ 2 for both single BCR B and dual BCR B cells was higher in the SLE group than in the HC group. The diversity of dual BCR B cells was lower in the SLE group than in the HC group (Figure 5E,F). The Gini index of both single BCR B and dual BCR B cells was higher in the SLE group than in the HC group (Figure 5G). No CDR3 overlap was found between dual BCR B cells in the SLE and HC groups, whereas partial CDR3 overlap existed between single BCR B and dual BCR B cells. Some of the partially shared CDR3 sequences originated from clonally expanded B cells, and the Jaccard overlap index was higher in the SLE group than in the HC group (Figure 5H). Single BCR B cells in the SLE group preferentially used *IGHV7* and *IGHJ3* genes, whereas dual BCR B cells in the SLE group preferentially used *IGHV3-33*, *IGHV3-48*, and *IGHV4-34* genes (Supplementary Figure S9B).

**Figure 6:**
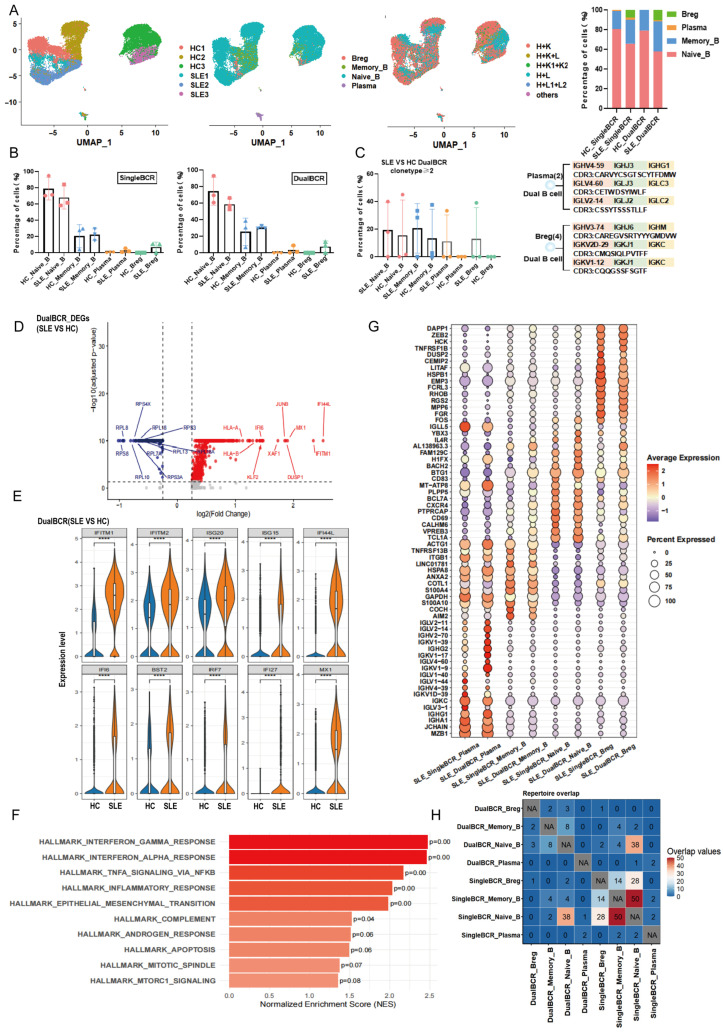
scRNA + BCR-seq analysis of the subset origins and characteristics of single BCR B cells and dual BCR B cells in peripheral blood of SLE patients and healthy controls. (**A**) Proportions and distributions of four B cell subpopulations in SLE patients and healthy controls; proportional distribution of the four B cell subpopulations; proportions and distributions of BCR pairing types within the four B cell subpopulations. (**B**) Proportions of single BCR B cells and dual BCR B cells within the four B cell subpopulations in SLE patients and healthy controls. (**C**) Clonal expansion proportions (clone type count ≥ 2) of dual BCR B cell subpopulations in the SLE group and the HC group; display of sequences from clonally expanded cells. (**D**) Volcano plot of differentially expressed genes in dual BCR B cells comparing the SLE group with the HC group. (**E**) Differential expression analysis of interferon pathway-related genes in dual BCR B cells between the SLE group and the HC group. (**F**) GSEA pathway enrichment analysis of dual BCR B cells comparing the SLE group with the HC group. (**G**) Comparison of the top 10 mRNA expressions of single and dual BCR B cell subpopulations in SLE patients. (**H**) Number of overlapping IGH CDR3 sequences among single and dual BCR B cell subpopulations in SLE patients. (**** *p* < 0.0001).

Subpopulation distribution of single BCR B cells: SLE group: Naive_B (68.02% ± 13.58%) > Memory_B (22.28% ± 7.22%) > Breg (6.99% ± 5.94%) > Plasma (2.71% ± 2.31%). HC group: Naive_B (78.88% ± 13.58%) > Memory_B (20.36% ± 14.32%) > Plasma (0.76% ± 0.47%). Subpopulation distribution of dual BCR B cells: SLE group: Naive_B (58.51% ± 6.95%) > Memory_B (30.54% ± 1.77%) > Breg (7.45% ± 6.74%) > Plasma (3.49% ± 4.74%). HC group: Naive_B (74.42% ± 16.82%) > Memory_B (25.36% ± 16.97%) > Plasma (0.19% ± 0.32%) (Breg not detected or below threshold). Compared with the HC group, dual BCR Naive_B cells were decreased in the SLE group, whereas dual BCR Memory_B, Breg, and Plasma cells were increased (Figure 6A,B). Cells with clone type count ≥ 2 in dual BCR B cell subpopulations in the SLE and HC groups: SLE_Naive_B (19.3% ± 20.13%) > HC_Naive_B (15.55% ± 24.17%); SLE_Memory_B (20.57% ± 18.21%) > HC_Memory_B (13.45% ± 20.43%). In the SLE group, dual BCR Plasma and Breg cells were 11.11% ± 19.24% and 13.02% ± 22.54%, respectively, whereas no clonal expansion was observed in the HC group (Figure 6C). Cells with clone type count ≥ 2 in single BCR B cell subpopulations in the SLE and HC groups: SLE_Naive_B (25.13% ± 24.0%) > HC_Naive_B (16.25% ± 25.58%); SLE_Memory_B (22.08% ± 22.10%) > HC_Memory_B (19.24% ± 22.03%); SLE_Plasma (40.01% ± 39.03%) > HC_Plasma (25.36% ± 43.93%). The clonal expansion proportion of SLE single BCR Breg cells was 15.41% ± 26.70%, whereas no clonal expansion was observed in the HC group (Supplementary Figure S10A,B,D).

Differential gene expression: In the SLE group, dual BCR B cells up-regulated genes such as MX1 and IFI6, and down-regulated genes such as *RPS8*, *RPS4X*, and *RPL18* (Figure 6D). In the SLE group, single BCR B cells up-regulated genes such as *IRF7*, *IFIT3*, and *IFI44*, and down-regulated genes such as *RPS2*, *FCER2*, and *NME2* (Supplementary Figure S11A). Dual BCR B cells in the SLE group expressed interferon-related genes including *IFITM1*, *IFITM2*, *ISG20*, *ISG15*, *IFI6*, *IFI27*, *MX1*, and *BST2* at significantly higher levels than those in the HC group (Figure 6E). Single BCR B cells in the SLE group also expressed these interferon-related genes (*IFITM1*, *IFITM2*, *ISG20*, *ISG15*, *IFI6*, *IFI27*, *MX1*, *BST2*) at significantly higher levels than those in the HC group (Supplementary Figure S11C). GSEA: In the SLE group, dual BCR B cells significantly enriched immune-inflammation-related pathways, including INTERFERON_GAMMA_RESPONSE, INTERFERON_ALPHA_RESPONSE (interferon signaling pathways), and TNFA_SIGNALING_VIA_NFKB (TNF inflammatory signaling pathway), whereas cell cycle pathways such as MITOTIC_SPINDLE were down-regulated (Figure 6F). In the SLE group, single BCR B cells also significantly enriched immune-inflammation-related pathways, including INTERFERON_ALPHA_RESPONSE, INTERFERON_GAMMA_RESPONSE (interferon signaling pathways), and TNFA_SIGNALING_VIA_NFKB (TNF inflammatory signaling pathway), whereas cell cycle pathways such as G2M_CHECKPOINT were down-regulated (Supplementary Figure S11D).

In the SLE group, the top 10 mRNA expressions of single BCR B and dual BCR B cell subpopulations were largely consistent, both expressing interferon-related genes such as *IFITM1*, *IFI6*, and *IFI44L* (Figure 6G and Figure S10C). In the SLE group, dual BCR Breg cells highly expressed *IL10* and *HSPA1A* (Supplementary Figure S11B). In the HC group, the top 10 mRNA expressions among single BCR B and dual BCR B cell subpopulations were homogeneous (Figure 6G and Figure S10C). Shared CDR3 sequences existed between single BCR B and dual BCR B cell subpopulations in both the SLE and HC groups, with a greater number of shared CDR3 sequences in the SLE group (Figure 6H and Figure S10E).

### 3.4. IGH Isotypes of Single BCR B and Dual BCR B Cells in SLE

IGH isotypes of dual BCR B cells in the MRL and H groups: IGHA: MRL_DualBCR (2.72% ± 0.50%) > H_DualBCR (0.32% ± 0.56%); IGHD: H_DualBCR (29.56% ± 4.80%) > MRL_DualBCR (2.17% ± 2.24%); IGHM: H_DualBCR (68.38% ± 6.72%) > MRL_DualBCR (30.65% ± 20.05%); IGHG: MRL_DualBCR (64.47% ± 22.44%) > H_DualBCR (1.74% ± 1.42%). IGH isotypes of single BCR B cells in the MRL and H groups: IGHA: MRL_SingleBCR (4.51% ± 1.82%) > H_SingleBCR (2.23% ± 0.38%); IGHD: H_SingleBCR (31.82% ± 6.06%) > MRL_SingleBCR (4.00% ± 2.48%); IGHM: H_SingleBCR (64.75% ± 5.67%) > MRL_SingleBCR (29.96% ± 8.50%); IGHG: MRL_SingleBCR (61.52% ± 12.22%) > H_SingleBCR (1.20% ± 0.35%). Both single and dual BCR B cells in the MRL group expressed significantly lower levels of IGHM and IGHD isotypes compared with the H group, but highly expressed IGHG, which is associated with autoantibodies (Supplementary Figure S14A).

IGH isotypes of dual BCR B cells in Groups B and C: IGHA: B_DualBCR (0.43%) > C_DualBCR (0.09%); IGHD: B_DualBCR (29.78%) > C_DualBCR (12.90%); IGHM: C_DualBCR (86.54%) > B_DualBCR (56.52%); IGHG: B_DualBCR (13.30%) > C_DualBCR (0.48%). IGH isotypes of single BCR B cells in Groups B and C: IGHA: B_SingleBCR (1.07%) > C_SingleBCR (0.14%); IGHD: B_SingleBCR (24.21%) > C_SingleBCR (15.59%); IGHM: C_SingleBCR (83.84%) > B_SingleBCR (51.39%); IGHG: B_SingleBCR (23.32%) > C_SingleBCR (0.43%). The proportion of IGHA in dual BCR B cells of Group B was higher than that in single BCR B cells of the same group and than that in the corresponding subpopulation of Group C. The proportion of IGHD in dual BCR B cells was also higher in Group B than in Group C (whereas the proportion of IGHD was higher in single BCR B cells of Group C). The proportion of IGHM in both single and dual BCR B cells of Group B was significantly higher than that in Group C, and the proportion of IGHG in dual BCR B cells of Group B was significantly higher than that in Group C (Supplementary Figure S14B).

IGH isotypes of dual BCR B cells in the SLE and HC groups: IGHA: SLE_DualBCR (5.57% ± 3.01%) > HC_DualBCR (3.81% ± 3.00%); IGHD: HC_DualBCR (18.40% ± 23.00%) > SLE_DualBCR (18.32% ± 22.17%); IGHM: HC_DualBCR (75.57% ± 19.47%) > SLE_DualBCR (69.18% ± 20.34%); IGHG: SLE_DualBCR (6.93% ± 0.97%) > HC_DualBCR (2.22% ± 1.62%). IGH isotypes of single BCR B cells in the SLE and HC groups: IGHA: SLE_SingleBCR (6.48% ± 3.40%) > HC_SingleBCR (4.73% ± 3.61%); IGHD: HC_SingleBCR (17.82% ± 22.58%) > SLE_SingleBCR (17.12% ± 20.09%); IGHM: HC_SingleBCR (73.36% ± 18.08%) > SLE_SingleBCR (65.76% ± 17.09%); IGHG: SLE_SingleBCR (10.64% ± 1.63%) > HC_SingleBCR (4.09% ± 2.70%). The proportions of IGHA and IGHM in both dual BCR B cells and single BCR B cells in the SLE group were higher than those in the HC group. The proportion of IGHD in dual BCR B cells was lower in the SLE group than in the HC group (whereas the proportion of IGHD in single BCR B cells was higher in the SLE group). The proportion of IGHG in dual BCR B cells was significantly higher in the SLE group than in the HC group, and the proportion of IGHG in single BCR B cells was also higher in the SLE group than in the HC group (Supplementary Figure S14C).

## 4. Discussion

The specificity, diversity, and memory of B cell responses to antigens are based on the clonal selection theory, which states that “one lymphocyte expresses only one type of antigen receptor” [4]. However, incomplete V(D)J allelic exclusion rearrangement can lead to the existence of “dual-receptor lymphocytes.” In recent years, dual BCR B cells have been confirmed to be closely associated with B cell-related tumors, aging and development, as well as the onset and progression of autoimmune diseases [5,6,7,8,9,10,15,16,17,18]. As a prototypical autoimmune disease, the detailed pathogenesis of SLE has not yet been fully elucidated, and dual BCR B cells may be involved in the initiation and progression of SLE [11,12]. However, due to technical limitations, the exact proportion, transcriptomic characteristics, and dynamic relationship of dual BCR B cells with single BCR B cells in SLE have not been fully elucidated. In this study, we performed a preliminary analysis of the proportion and characteristics of dual BCR B cells in systemic lupus erythematosus (SLE) model mice and SLE patients using scRNA-seq and scBCR-seq technologies.

### 4.1. Proportion and CDR3 Repertoire Characteristics of Dual BCR B Cells in SLE Model Mice and SLE Patients

In SLE model mice and SLE patients, the proportion of dual BCR B cells showed a decreasing trend compared with healthy controls, which is largely consistent with the findings of Peterson et al., who reported that “only one quarter of SLE patients have a high frequency of dual BCR B cells” [13], but opposite to the results reported by Fraser et al. [11] and Sang et al. [12], Possible significance and reasons: (1) Compared with healthy controls, the degree of clonal expansion of dual BCR B cells was higher in the SLE patient group, the diversity of the CDR3 repertoire was reduced, and the unevenness of clonal distribution (increased Gini index) was more pronounced, suggesting that dual BCR B cells may be involved in the specific autoimmune response in SLE. (2) The Jaccard index for shared CDR3 sequences between single BCR B and dual BCR B cells was higher in the SLE patient group than in the healthy control group, suggesting that they may originate from the same progenitor cells and may participate in responses to the same antigens. Similar findings have been reported in studies of dual TCR T cells and non-small cell lung cancer [19]. (3) The dual BCR B cells included in this experiment comprised dual-light-chain (Hκκ, Hλλ, Hκλ) B cells, dual-heavy-chain (HHκ, HHλ) B cells, as well as dual-heavy-chain and dual-light-chain (HHκλ, HHκκ, HHλλ, etc.) B cells. The total cell counts were calculated as proportions based on high-throughput single B cells (at the mRNA level). In previous studies, the definition of dual BCR B cells was mainly based on Igκ + λ protein expression levels, and the total number of B cells included for analysis was limited, which may account for the inconsistency in the reported proportions of dual BCR B cells. The pairing types and patterns of H, κ, and λ of dual BCR B cells first defined at the mRNA level in this study provide novel insights for in-depth research on dual BCR B cells in SLE; however, the proportion, effects, and functions of dual BCR B cells need to be verified at the BCR protein expression level.

Under the pathological condition of SLE, single BCR B cells and dual BCR B cells exhibit inconsistent high-frequency usage of V and J genes. Dual BCR B cells in SLE model mice and SLE patients also show different VJ gene usage preferences, suggesting that they may participate in the diversity of antigenic epitope recognition and differential responses. Compared with the healthy group, dual BCR B cells in SLE patients highly express the *IGHV4-34* gene, which has been repeatedly reported to be closely associated with the progression of SLE [20,21], indirectly suggesting that dual BCR B cells may be involved in the initiation and development of SLE. Furthermore, partial CDR3 overlap exists between clonally expanded single BCR B cells and dual BCR B cells in both SLE model mice and SLE patients, which is similar to our findings in dual TCR T cell studies [19,22,23], suggesting that they may recognize common antigenic epitopes and jointly participate in the autoimmune response in SLE.

In the analysis of immunoglobulin isotypes of single BCR B cells and dual BCR B cells from SLE patients, compared with the healthy group, the SLE group showed an increased proportion of the IGHG isotype and a decreased proportion of IGHM. Similar characteristics were also observed in the two model mouse strains. Since a high proportion of the IGHG isotype is closely associated with autoimmune responses in SLE [24], this finding suggests that further investigation into the proportion, origin, characteristics, and effector functions of IGHG isotype B cells in SLE patients is warranted.

### 4.2. Differential Gene Expression and Gene Enrichment Pathways of Dual BCR B Cells in SLE Model Mice and SLE Patients

Shmerling et al. have demonstrated that the *Ly6a* and *Ly6c2* genes are associated with interferon induction and participate in the initiation and progression of SLE [25,26,27,28]. A breakthrough advantage of scRNA + BCR-seq technology is its ability to track and analyze, at the high-throughput single-B-cell level, the dynamic relationship between the transcriptome of individual B cell subpopulations and BCR clone types [15]. In the two SLE model mice, we found that dual BCR B cells in the disease groups highly expressed interferon-related genes such as *Ly6a*, *Ly6c2*, *Jchain*, *Isg20*, and *Ifi27* compared with the healthy groups, suggesting that dual BCR B cells participate in the initiation and progression of SLE via the interferon pathway. GSEA of the two SLE model mice revealed that in MRL/Lpr mice, single BCR B cells were significantly enriched in a series of pathways related to inflammatory activation, cellular stress, and metabolic reprogramming, indicating that single BCR B cells reside in a highly active pro-inflammatory and stress microenvironment with enhanced proliferative and responsive capacity. In contrast, the most prominent feature of dual BCR B cells was profound inhibition of cell-cycle-related pathways, manifested by significant negative enrichment of the MITOTIC_SPINDLE pathway, suggesting that while single BCR B cells tend toward proliferation and effector differentiation, dual BCR B cells may enter a relatively quiescent, terminally differentiated, or functionally distinct immunoregulatory state. Notably, in the SLE.Yaa model, dual BCR B cells significantly enriched the type I interferon response pathway (INTERFERON_ALPHA_RESPONSE), which is a core pathogenic feature of this model, where Tlr7 gene translocation leads to enhanced TLR7 signaling and induction of type I interferon production [29,30], The above pathway enrichment results are consistent with the genetic background of the models, suggesting that dual BCR B cells may be more sensitive to activation of the TLR7-interferon axis. In contrast, the core feature of the MRL/lpr model is a defect in the Fas-mediated apoptosis pathway [31,32], dual BCR B cells did not show significant enrichment of interferon pathways, but instead exhibited inhibition of cell cycle-related pathways. Dual BCR B cells exhibited different functional states (quiescent vs. highly activated interferon signature) in the two models, suggesting a possible link to the distinct pathogenic mechanisms of the SLE models (e.g., TLR7-IFNα or Fas-FasL). In SLE patients, we also found that both single BCR B and dual BCR B cells highly expressed interferon-related genes. Multiple studies have shown that the interferon-related genes *IFI44L* and *MX1* serve as biomarkers for SLE in blood-based studies, and that *MX1* has demonstrated strong diagnostic potential in proteomics and single-cell sequencing studies [33,34,35,36,37,38,39,40,41]. In our volcano plot differential expression analysis, we found that dual BCR B cells highly expressed *IFI44L* and *MX1*, whereas single BCR B cells highly expressed *IFI44L*. Compared with healthy controls, dual BCR B cells from SLE patients exhibited high expression of *MX1*. Whether this can serve as a characteristic diagnostic marker for dual BCR B cells in SLE remains to be further investigated. Re-analysis of interferon pathway gene expression in single BCR B and dual BCR B cells from SLE patients revealed that both single BCR B and dual BCR B cells exhibited a strong “interferon signature.” GSEA showed co-enrichment of both type I interferon (INTERFERON_ALPHA_RESPONSE) and type II interferon (INTERFERON_GAMMA_RESPONSE) pathways. Furthermore, single BCR B and dual BCR B cells shared core inflammatory pathways such as TNFA_SIGNALING_VIA_NFKB and INFLAMMATORY_RESPONSE, suggesting their joint participation in the highly inflammatory and interferon-driven immune environment characteristic of the SLE pathological state. Notably, dual BCR B cells in SLE patients showed even higher enrichment of interferon response pathways, potentially positioning them at the center of interferon signaling. In 2018, Allison Sang et al. also demonstrated that these cells exhibit stronger responses to Toll-like receptors and type I/II interferons [12]. This suggests that in previous studies of interferon-related genes and inflammatory pathways in B cells from SLE patients, the potential involvement of dual BCR B cells has been overlooked, and their identification may provide a novel B cell subpopulation for further in-depth investigation.

Meanwhile, abnormal activation of the complement system is one of the core pathological features of SLE [42]. In SLE patients, we found that dual BCR B cells significantly enriched the COMPLEMENT pathway, suggesting that dual BCR B cells may play a more direct role in tissue damage and immune complex clearance, either through antibody production or direct involvement in the complement cascade. Furthermore, defective clearance of apoptotic cells is an important source of self-antigens in SLE [42,43]. We found that dual BCR B cells from SLE patients enriched the APOPTOSIS pathway, suggesting that dual BCR B cells are more sensitive to apoptotic signals or interact more frequently with apoptosis-derived self-antigens, thereby exacerbating the autoimmune response.

### 4.3. Proportions of Dual BCR B Cell Subpopulations and Their Gene Expression Characteristics in SLE Model Mice and SLE Patients

Compared with healthy controls, in SLE model mice and SLE patients, the proportion of naive B cells was decreased while the proportion of plasma cells was increased under the pathological condition of SLE, which is consistent with previous reports [44,45]. In the present study, further analysis of single BCR B cells and dual BCR B cells revealed that the subpopulation distributions of the two were highly consistent. We have also observed a similar trend in the subpopulation distribution of single TCR T cells and dual TCR T cells in pemphigus [23]. Significant clonal expansion was observed in dual BCR plasma cells in SLE, and there were differences in the preferential V gene usage between single BCR plasma cells and dual BCR plasma cells. For example, in MRL/Lpr mice, single BCR plasma cells highly expressed genes such as *Ighv1-5* and *Igkv1-7*, whereas dual BCR plasma cells highly expressed *Ighv1-85* and *Igkv6-23*, suggesting that the two may recognize different antigenic epitopes and cooperatively participate in the autoimmune response.

The top 10 mRNA expressions in single BCR B and dual BCR B cell subpopulations were largely consistent, suggesting that single BCR B cells and dual BCR B cells participate in the same immune responses, which is similar to our findings on dual TCR T cells in pemphigus and nasopharyngeal carcinoma [22,23,46]. Breg cells are a subset of B cells with immunomodulatory functions. They participate in immune regulation in various diseases such as autoimmune diseases, infections, and tumors, and are able to suppress inflammatory responses by secreting anti-inflammatory cytokines such as IL-10 [47]. In this study, we observed an increasing trend of Breg cells in both SLE model mice and SLE patients, consistent with previous reports [48]. Iwata et al. (2010) found that some untreated patients with pemphigus, lupus, and rheumatoid arthritis also exhibited an increased frequency of Breg cells [49]. The increased frequency of Breg cells may be related to the severe autoimmune inflammation the body is facing; the body initiates a “compensatory regulation” by expanding IL-10-expressing Breg cells in an attempt to suppress inflammation and avoid tissue damage. The increased frequency of Breg (IL-10) cells is consistent with the levels observed during inflammation [50,51,52,53]. In the MRL/Lpr mouse model, we found that dual BCR Breg cells highly expressed genes such as *HSPA1A* and *Hspa5* (the Hsp family may participate in the functional regulation of Breg cells, e.g., *Hsp70* enhances the suppressive function of Breg cells), as well as higher expression of IL-10 [54,55]. In SLE patients, we also found that dual BCR Breg cells highly expressed *IL10* and *HSPA1A* (Supplementary Figure S11B), suggesting that they are in an activated state, which may facilitate their involvement in antigen presentation and regulation of autoimmune responses.

This study takes full advantage of the groundbreaking capability of single-cell RNA + VDJ-seq technology to resolve dual BCR B cells at the high-throughput single-B-cell level. We have preliminarily characterized the proportion, CDR3 repertoire features, and subset origin distribution of dual BCR B cells in SLE model mice and SLE patients. Furthermore, based on BCR CDR3 receptor clone types, we compared and tracked the similarities and differences in the expression of highly expressed effector molecules between single BCR B cells and dual BCR B cells at the mRNA transcriptome level. The findings and analytical framework of this study may provide baseline comparative data and novel research perspectives for further in-depth investigation of B cell effector functions and pathogenic mechanisms in SLE patients. However, it is important to emphasize that this study is limited by a small sample size and the inability to perform subgroup analyses. Moreover, while spleen samples were analyzed from SLE model mice, peripheral blood samples were analyzed from SLE patients. Due to species-specific differences in B cells and variations in B cell distribution across different tissue compartments, a direct comparative analysis of B cell differences between the two is theoretically not feasible. The technical approach of this study relies primarily on single-cell RNA + VDJ-seq technology, which examines biological phenomena at the mRNA level, and there remain unknown technical issues concerning the ability of this technology to accurately detect “dual-receptor lymphocytes”. Therefore, the relationship between SLE and dual BCR B cells warrants further investigation using larger sample sizes, with subgroup analyses based on SLE flare and remission phases as well as pre-treatment and post-treatment status. Most notably, validation studies on the effector functions and underlying mechanisms of dual BCR B cells at the protein expression level are urgently needed. At present, because the IGκ and IGλ loci in humans and mice are located on different chromosomes, monoclonal antibodies against the constant regions of the κ chain and λ chain can be generated. Using distinct fluorescent labels, these antibodies enable the proportional analysis of “κ + λ”-positive “dual BCR B cells.” However, it is currently not possible to generate monoclonal antibodies that distinguish the constant regions of the two chromosome-encoded chains of the H chain (κ chain or λ chain); consequently, flow cytometry cannot be directly used to analyze “dual-heavy-chain,” “dual-κ-chain,” or “dual-λ-chain” dual BCR B cells. The successful generation and application of the “dual TCR alpha chain fluorescent reporter mouse” [56,57] provides a paradigm for further studies of dual BCR B cells.

## Data Availability

The single-cell sequencing data generated in this study for MRL/Lpr mice and healthy C57BL/6 mice have been deposited in the NCBI database under the accession number PRJNA1413971 (https://www.ncbi.nlm.nih.gov/bioproject/PRJNA1413971 (accessed on 25 January 2026)). The single-cell sequencing data of SLE patients, SLE.Yaa model mice, and corresponding healthy controls used in this study were all obtained from public databases (Gene Expression Omnibus). The single-cell RNA sequencing (scRNA-seq) and single-cell B-cell receptor sequencing (scBCR-seq) data are available in the Gene Expression Omnibus under accession numbers GSE192762 and GSE193867, respectively. All data in these databases are publicly available under an open-access format, and no authorization or approval from any institution or regulatory authority is required for their use. The code used in this study can be obtained from the corresponding author upon reasonable request.

## Supplementary Materials

The following supporting information can be downloaded at: https://www.mdpi.com/article/10.3390/cells15100914/s1, Figure S1: Sample preparation from MRL/lpr and healthy C57BL/6 mice for single-cell sequencing and immunohistochemistry; Figure S2: Flow cytometric analysis of the proportion of κ^+^λ^+^ B cells in the spleens of C57BL/6 mice (*n* = 6) and MRL mice (*n* = 4); Figure S3: IGHV subfamily usage proportions and clonal sequence overlap from scBCR-seq data analysis in MRL/lpr model and control mice; Figure S4: scRNA-seq and scBCR-seq analysis of subset characteristics of single- and dual-BCR B cells in the spleens of healthy (H group) control mice; Figure S5: scRNA-seq and scBCR-seq analysis of characteristics of single- and dual-BCR B cells in the spleens of MRL and H group mice; Figure S6: Analysis of V and J gene usage and clonal sequence overlap from scBCR-seq data in SLE.Yaa model mice (Group B) and control mice (Group C); Figure S7: scRNA-seq and scBCR-seq analysis of subset characteristics of single- and dual-BCR B cells in the spleens of healthy control mice; Figure S8: Integrated scRNA-seq and scBCR-seq analysis of characteristics of single- and dual-BCR B cells in the spleens of SLE.Yaa model mice (Group B) and control mice (Group C); Figure S9: Analysis of V and J gene usage from scBCR-seq data in SLE patients (SLE group) and healthy volunteers (HC group); Figure S10: Integrated scRNA-seq and scBCR-seq analysis of subset characteristics of single- and dual BCR Bcells in human peripheral blood; Figure S11: Integrated scRNA-seq and scBCR-seq analysis of characteristics of single- and dual-BCR B cells in peripheral blood from the SLE and HC groups; Figure S12: Diversity analysis (inverse Simpson index) of single- and dual-BCR B cells across groups by integrated scRNA-seq and scBCR-seq; Figure S13: Jaccard analysis of clonal overlap for single- and dual-BCR B cells across groups by integrated scRNA-seq and scBCR-seq; Figure S14: Integrated scRNA-seq and scBCR-seq comparative analysis of immunoglobulin heavy chain (IGHA/IGHD/IGHM/IGHG) isotype distribution in single- and dual-BCR B cells from two SLE mouse models (MRL/lpr, SLE.Yaa) and SLE patients versus healthy controls.
