# Supplementary material for: Preliminary Analysis of the Proportion and Characteristics of Dual BCR B Cells in SLE Model Mice and Patients via scRNA-Seq Combined with scBCR-Seq Technology
Source: Cells. 2026 May 17;15(10):914. doi: 10.3390/cells15100914 (PMC13204964; doi:10.3390/cells15100914)
Supplement: Supplementary file 1 [file cells-15-00914-s001.zip › cells-4274300-supplementary.pdf]

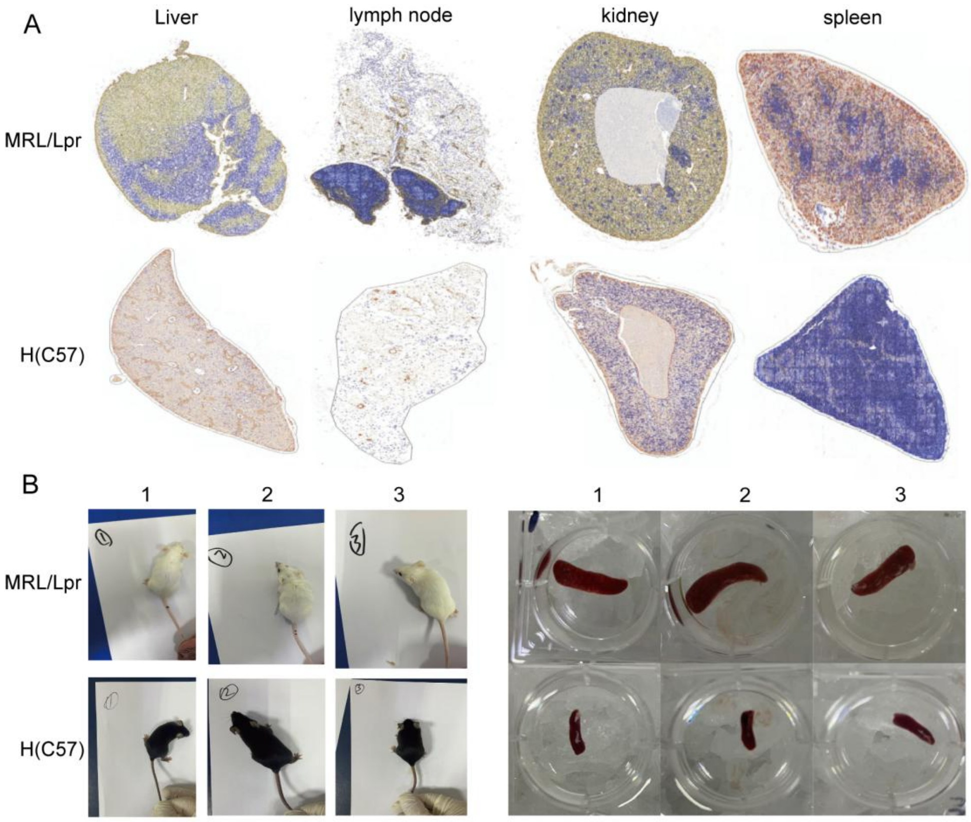

C

Sample	Sequencing volume	Average reads per cell	Barcode Q30(%)	Reads2 Q3 (%)	UMI Q30 (%)
H1	40732427	19186	91.2	85.9	92.6
H2	38161558	11375	91.5	85.8	93.2
H3	38590141	16153	91.2	86.2	93.3
MRL1	35806507	29227	96.2	94.0	97.2
MRL2	41871967	19512	96.4	94.0	97.4
MRL3	41882094	18681	96.3	94.1	97.4

D

Sample	Number of blast cells	Exclude doublet counts /percentages
MRL1	12457	1075/8.63%
MRL2	12646	1108/8.76%
MRL3	10452	745/7.13%
H1	9334	594/6.36%
H2	11854	987/8.33%
H3	9051	344/3.80%

Sup Fig S1. Sample preparation from MRL/lpr and healthy C57BL/6 mice for single-cell sequencing and immunohistochemistry.

A. Spleen tissues from three MRL/lpr mice (female, 13 weeks old) and three healthy C57BL/6 mice (female, 13 weeks old) were harvested for single-cell sequencing. B. Immunohistochemistry for CD19+ cells was performed on the spleen, lymph node, kidney, and liver from one MRL/lpr mouse and healthy mouse(female, 13 weeks old). C. Single-Cell Sequencing Sample Quality Inspection Report from OE Biotech Co., Ltd., Shanghai. D. Doublet Removal Rate of Single-Cell Sequencing Data in MRL Group and H Group Mice in This Study.

A

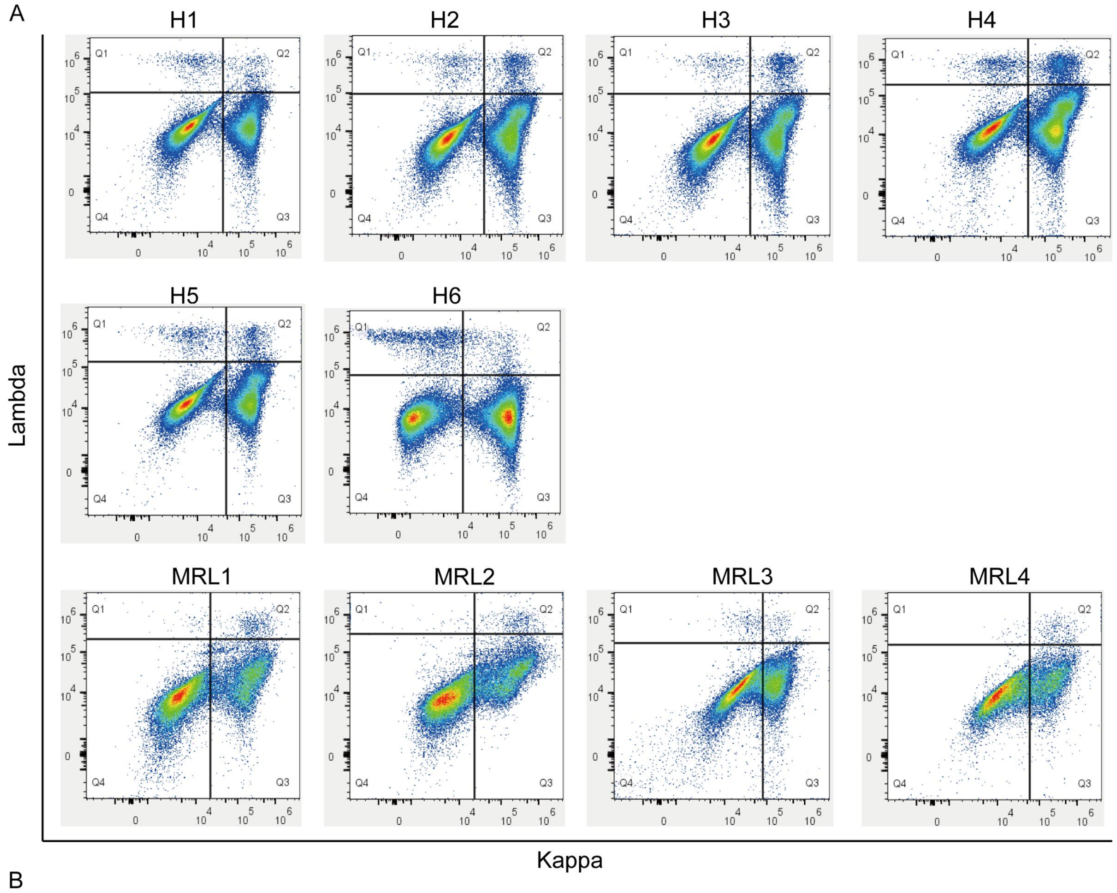

B

Mouse	H1	H2	H3	H4	H5	H6	S1	S2	S3	S4
Igκ$^+$Igλ$^+$(%)	2.40%	4.05%	4.27%	5.37%	3.28%	1.55%	4.54%	3.48%	4.19%	1.29%

Sup Fig S2. Flow cytometric analysis of the proportion of κ$^+$λ$^+$ B cells in the spleens of C57BL/6 mice(n=6) and MRL mice(n=4) .

A. Flow cytometric analysis of Igκ$^+$Igλ$^+$ B cells in spleen samples from the MRL group and H groupCells were stained with PE-conjugated anti-Igλ and FITC-conjugated anti-Igκ, followed by flow cytometric analysis. Dot plots show logarithmic fluorescence data of stained spleen cells after gating on lymphocytes based on side scatter (SSC) and forward scatter (FSC). B.Frequency of Igκ$^+$Igλ$^+$ B cells in spleen samples from mice in the MRL group and H group.

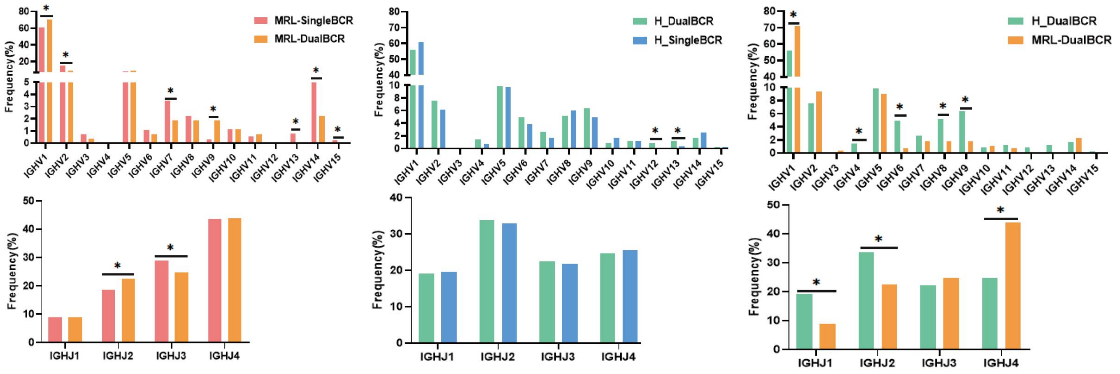

Sup Fig S3. IGHV subfamily usage proportions and clonal sequence overlap from scBCR-seq data analysis in MRL/lpr model and control mice. IGHV subfamily usage proportions in single- and dual-BCR B cells from the MRL group; IGHV subfamily usage proportions in single- and dual-BCR B cells from the H group; Comparison of IGHV subfamily usage proportions in dual-BCR B cells between the MRL and H groups (*P < 0.05).

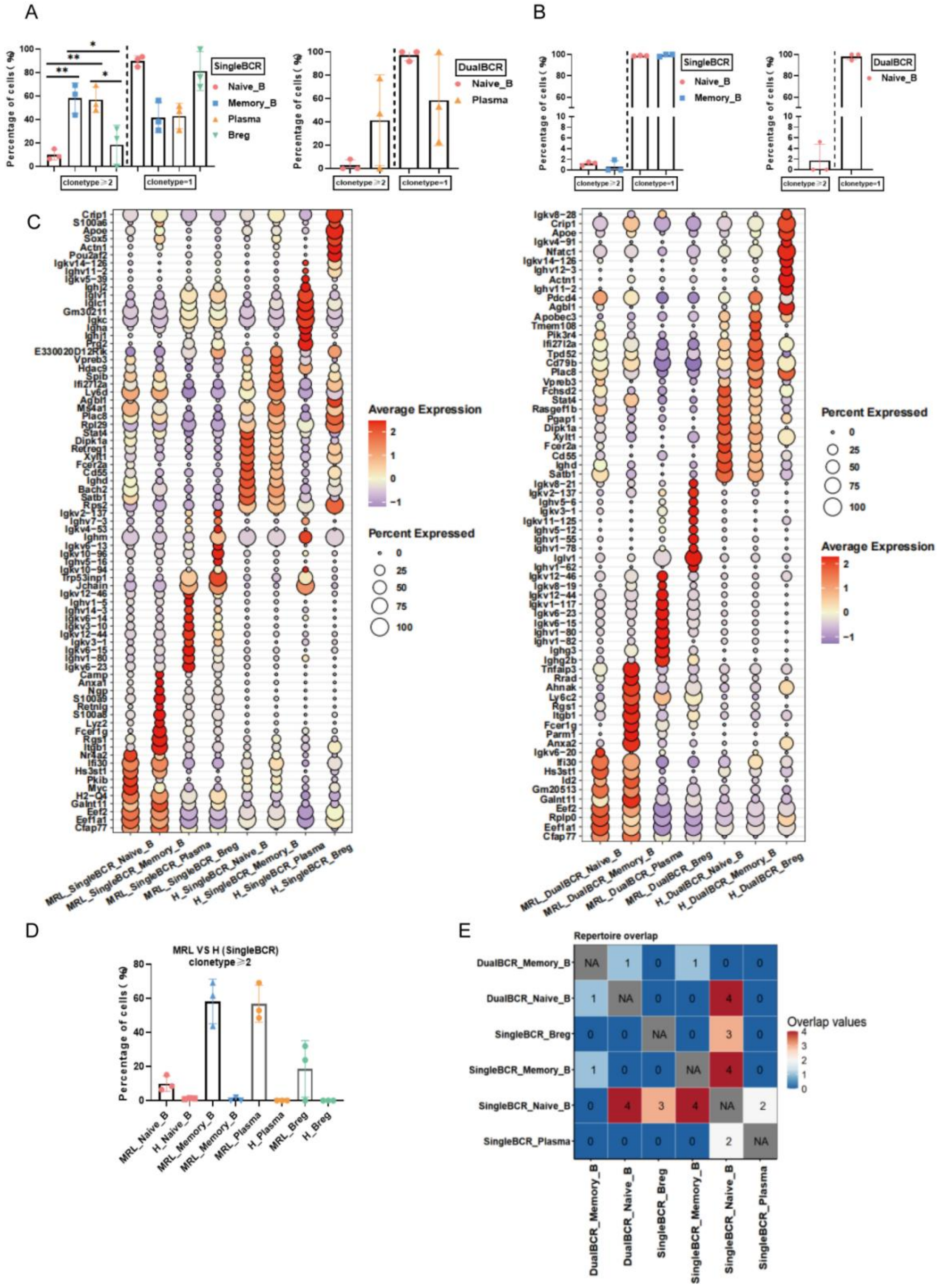

Sup Fig S4. scRNA-seq and scBCR-seq analysis of subset characteristics of single- and dual-BCR B cells in the spleens of healthy(H group) control mice.

A. Clonal expansion proportion (clone type ≥2 vs. clone type =1) in single-BCR B cell subsets from the MRL group. B. Clonal expansion proportion (clone type ≥2 vs. clone type =1) in single-BCR B cell subsets from the H group.C. Comparison of the top 1 highly expressed mRNAs across single- and dual-BCR B cell subsets in the MRL and H groups.D. Clonal expansion proportion (clone type ≥2) in single-BCR B cell subsets in the MRL and H groups.E. Number of overlapping IGH CDR3 sequences among single- and dual-BCR B cell subsets in the healthy control group.(*p＜.5，**p＜.1)

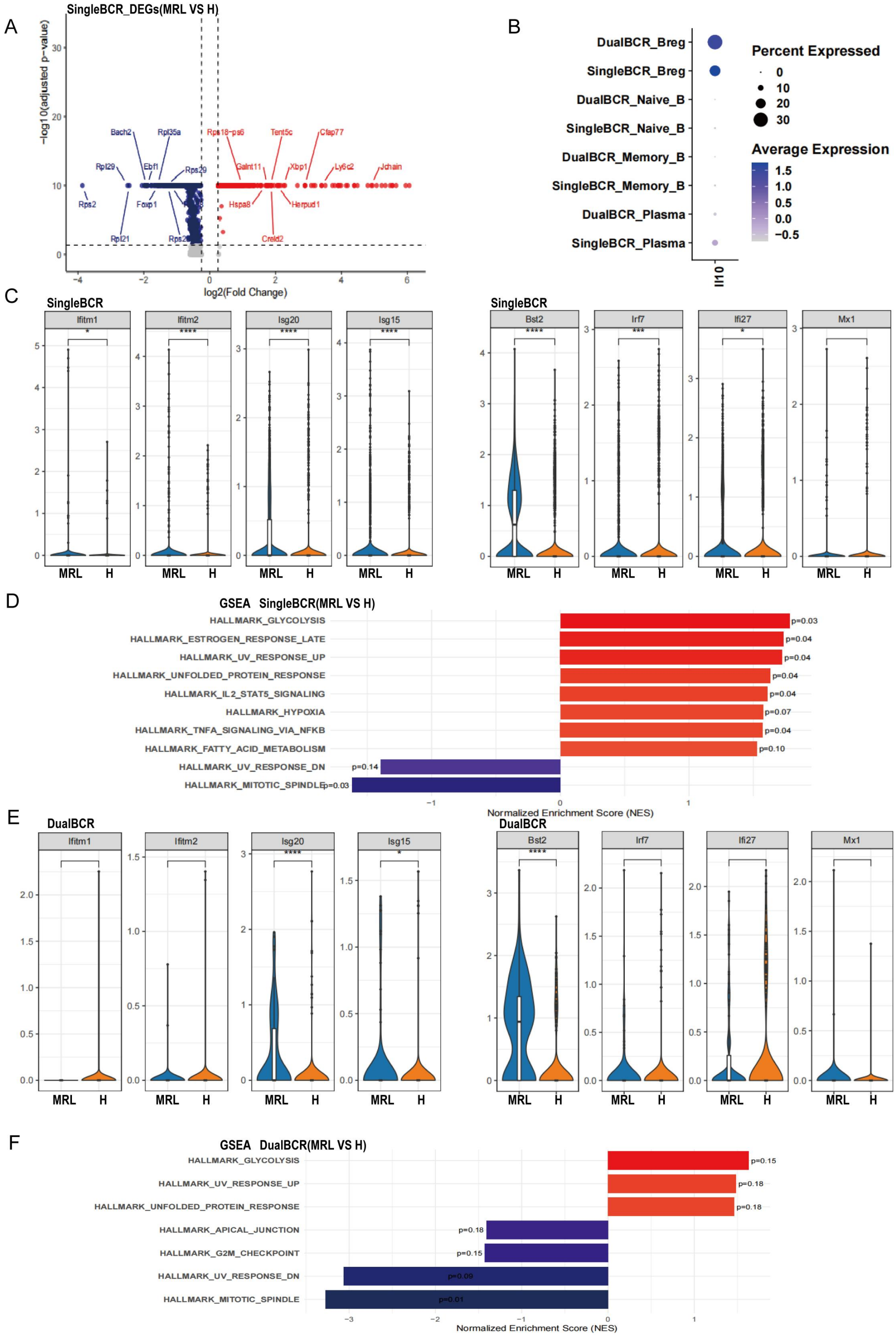

Sup Fig S5. scRNA-seq and scBCR-seq analysis of characteristics of single- and dual-BCR B cells in the spleens of MRL and H group mice.

A. Volcano plot of differentially expressed genes in dual-BCR B cells from the MRL group compared to the H group.B. Analysis of IL-1 expression across single- and dual-BCR B cell subsets in the MRL group.C. Differential expression analysis of interferon pathway-related genes in single-BCR B cells from the MRL and H groups.D. GSEA pathway enrichment analysis of single-BCR B cells from the MRL group compared to the H

group.E. Differential expression analysis of interferon pathway-related genes in dual-BCR B cells from the MRL and H groups.F. GSEA pathway enrichment analysis of dual-BCR B cells from the MRL group compared to the H group.(*p＜0.05，**p＜0.01，***p＜0.001，****p＜0.001)

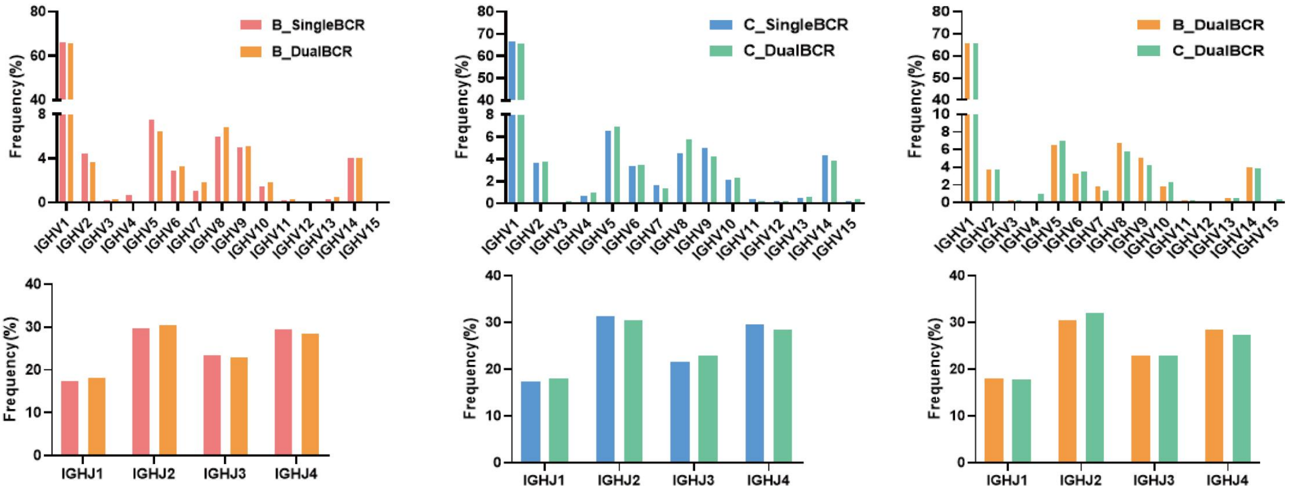

Sup Fig S6. Analysis of V and J gene usage and clonal sequence overlap from scBCR-seq data in SLE.Yaa model mice (Group B) and control mice (Group C). IGHV subfamily usage proportions in single- and dual-BCR B cells from Group B; IGHV subfamily usage proportions in single- and dual-BCR B cells from Group C; comparison of IGHV subfamily usage proportions in dual-BCR B cells between Groups B and C.

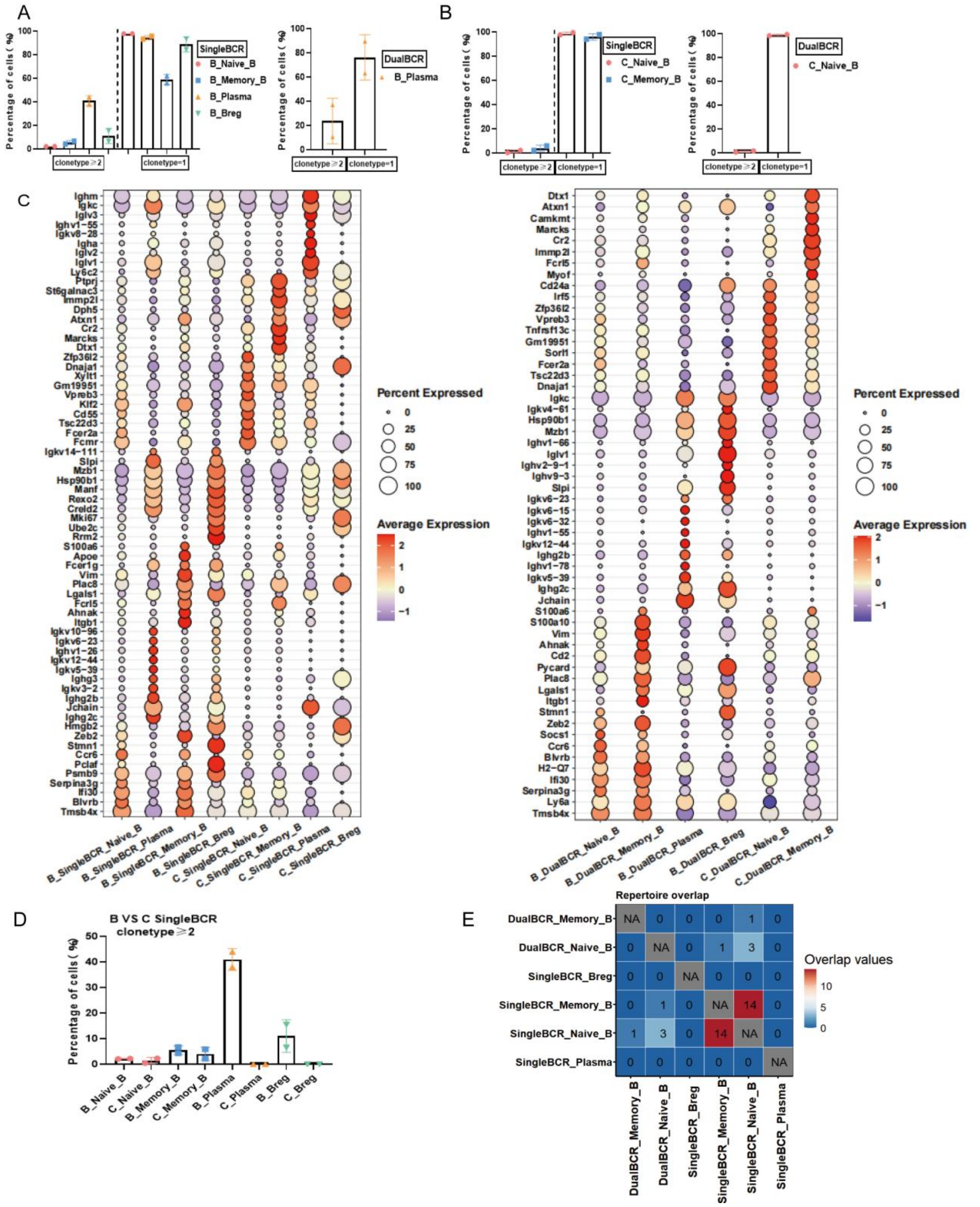

Sup Fig S7. scRNA-seq and scBCR-seq analysis of subset characteristics of single- and dual-BCR B cells in the spleens of healthy control mice.

A. Clonal expansion proportion (clone type ≥2 vs. clone type =1) in B cell subsets from Group B. B. Clonal expansion proportion (clone type ≥2 vs. clone type =1) in B cell subsets from Group C.C. Comparison of the top 10 highly expressed mRNAs across single- and dual-BCR B cell subsets in Groups B and C.D. Analysis of clonal expansion proportion (clone type ≥2) in single-BCR B cell subsets in Groups B and C.E. Number of overlapping IGH CDR3 sequences among single- and dual-BCR B cell subsets in Group C.

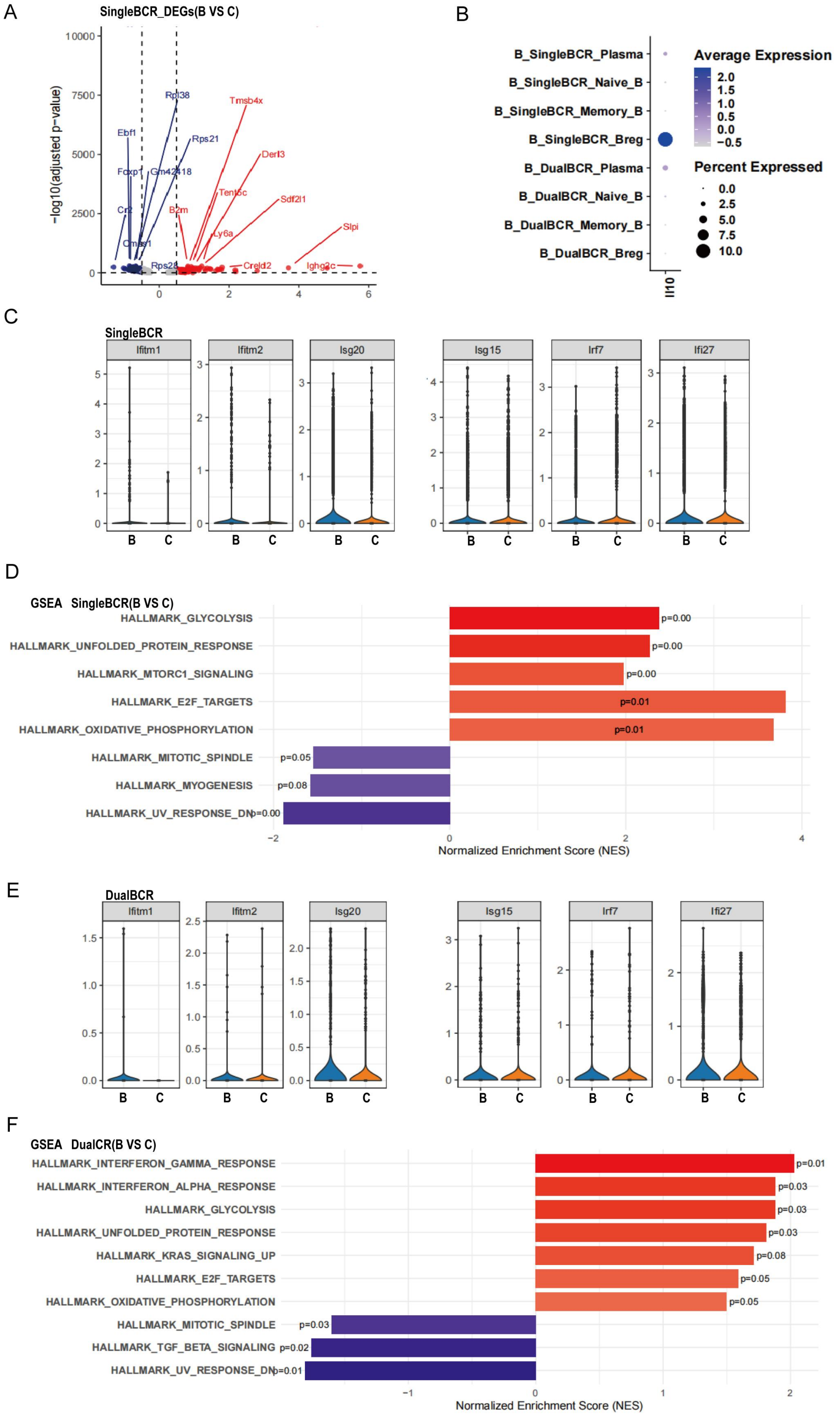
A
SingleBCR_DEGs(B VS C)
-log10(adjusted p-value)
Rpl38
Tmsb4x
Ebf1
Rps21
Derl3
Foxp1
Gm42418
Tent5c
Sdf2l1
Cr2
B2m
Slpi
Ly6a
Cmas1
Rps28
Creld2
Ighg2c
B
B_SingleBCR_Plasma
B_SingleBCR_Naive_B
B_SingleBCR_Memory_B
B_SingleBCR_Breg
B_DualBCR_Plasma
B_DualBCR_Naive_B
B_DualBCR_Memory_B
B_DualBCR_Breg
Il10
Average Expression
Percent Expressed
C
SingleBCR
Ifitm1
Ifitm2
Isg20
Isg15
Irf7
Ifi27
D
GSEA SingleBCR(B VS C)
HALLMARK_GLYCOLYSIS p=0.00
HALLMARK_UNFOLDED_PROTEIN_RESPONSE p=0.00
HALLMARK_MTORC1_SIGNALING p=0.00
HALLMARK_E2F_TARGETS p=0.01
HALLMARK_OXIDATIVE_PHOSPHORYLATION p=0.01
HALLMARK_MITOTIC_SPINDLE p=0.05
HALLMARK_MYOGENESIS p=0.08
HALLMARK_UV_RESPONSE_DN p=0.00
Normalized Enrichment Score (NES)
E
DualBCR
Ifitm1
Ifitm2
Isg20
Isg15
Irf7
Ifi27
F
GSEA DualCR(B VS C)
HALLMARK_INTERFERON_GAMMA_RESPONSE p=0.01
HALLMARK_INTERFERON_ALPHA_RESPONSE p=0.03
HALLMARK_GLYCOLYSIS p=0.03
HALLMARK_UNFOLDED_PROTEIN_RESPONSE p=0.03
HALLMARK_KRAS_SIGNALING_UP p=0.08
HALLMARK_E2F_TARGETS p=0.05
HALLMARK_OXIDATIVE_PHOSPHORYLATION p=0.05
HALLMARK_MITOTIC_SPINDLE p=0.03
HALLMARK_TGF_BETA_SIGNALING p=0.02
HALLMARK_UV_RESPONSE_DN p=0.01
Normalized Enrichment Score (NES)

Sup Fig S8. Integrated scRNA-seq and scBCR-seq analysis of characteristics of single- and dual-BCR B cells in the spleens of SLE.Yaa model mice (Group B) and control mice (Group C).

A. Volcano plot of differentially expressed genes in dual-BCR B cells from Group B compared to Group C.B. Analysis of IL10 expression across single- and dual-BCR B cell subsets in Group B.C. Differential expression analysis of interferon pathway-related genes in single-BCR B cells from Groups B and C.D. GSEA pathway enrichment analysis of single-BCR B cells from Group B compared to Group C.E. Differential expression analysis of interferon pathway-related genes in dual-BCR B cells from Groups B and C.F. GSEA pathway enrichment analysis of dual-BCR B cells from Group B compared to Group C.

A

Donor	Age	Sex	Ethnicity	Auto-antibodies	Tissue
HC1	34	F	Caucasian	--	Blood
HC2	43	F	Caucasian	--	Blood
HC3	42	F	African Caribbean	--	Blood
SLE 1	31	F	African Caribbean	ANA, dsDNA, Sm, RNP, C1Q	Blood
SLE 2	48	F	Caucasian	ANA, DNA, RNP	Blood
SLE 3	30	F	African Caribbean	ANA, Sm, RNP, La, C1Q & SLE, LN	Blood

B

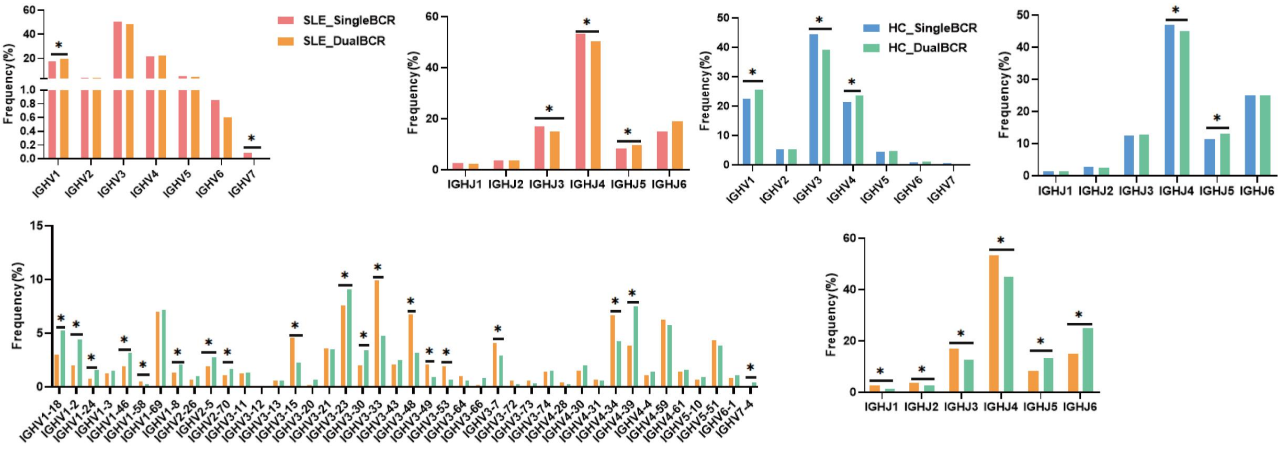

Sup Fig S9. Analysis of V and J gene usage from scBCR-seq data in SLE patients (SLE group) and healthy volunteers (HC group).

A. Baseline Characteristics of SLE Patients and Healthy Volunteers(Note: All information was obtained from the original data. Blood samples from SLE patients and healthy controls were collected with informed consent and REC approval. Approval number: 11/LO/1433). B. IGHV subfamily usage proportions in single- and dual-BCR B cells from the SLE group; IGHV subfamily usage proportions in single- and dual-BCR B cells from the healthy control (HC) group; comparison of IGHV subfamily usage proportions in dual-BCR B cells between the SLE and HC groups.(*$p < 0.05$)

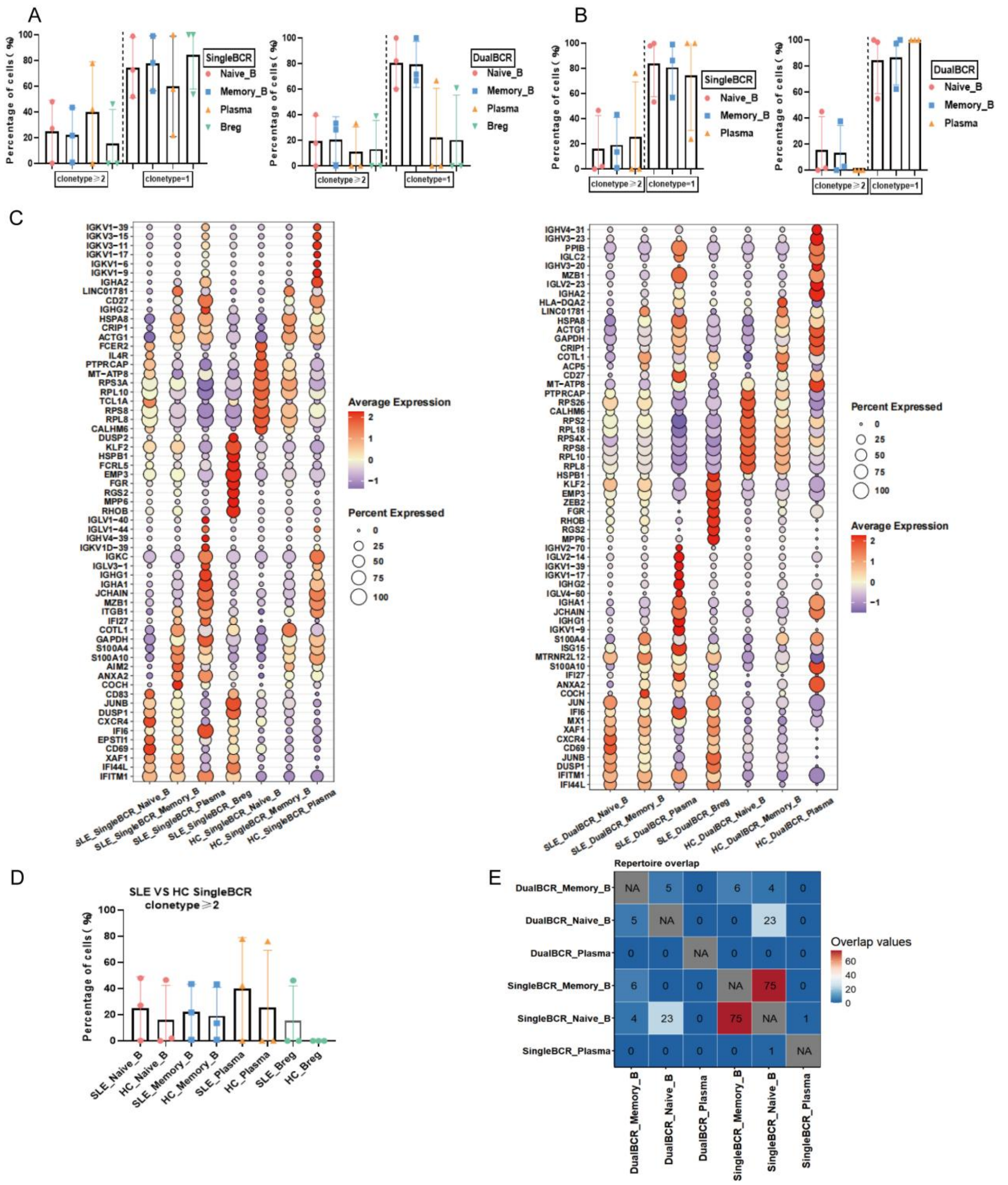

Sup Fig S10. Integrated scRNA-seq and scBCR-seq analysis of subset characteristics of single- and dual-BCR B cells in human peripheral blood.

A. Clonal expansion proportion (clone type ≥2 vs. clone type =1) in single- and dual-BCR B cell subsets from the SLE patient group. B. Clonal expansion proportion (clone type ≥2 vs. clone type =1) in single- and dual-BCR B cell subsets from the healthy control (HC) group. C. Comparison of the top 10 highly expressed mRNAs across single- and dual-BCR B cell subsets in the SLE and HC groups. D. Proportional analysis of clonal expansion (clone type ≥2) in single-BCR B cell subsets in the SLE and HC groups. E. Number of overlapping IGH CDR3 sequences among single- and dual-BCR B cell subsets in SLE patients and healthy volunteers.

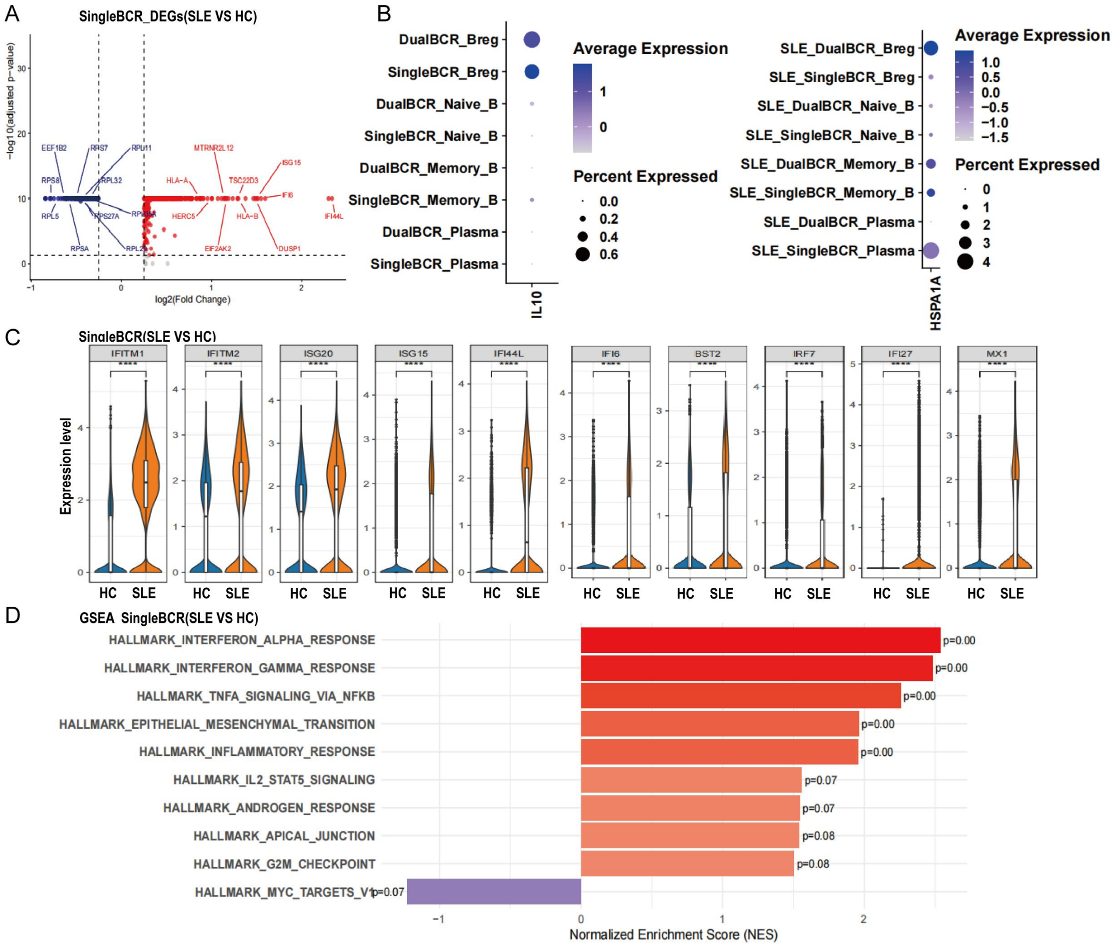

Sup Fig S11. Integrated scRNA-seq and scBCR-seq analysis of characteristics of single- and dual-BCR B cells in peripheral blood from the SLE and HC groups.

A. Volcano plot of differentially expressed genes in single-BCR B cells from the SLE group compared to the HC group. B. Differential expression analysis of IL10 and HSPA1A across single- and dual-BCR B cell subsets in the SLE group. C. Differential expression analysis of interferon pathway-related genes in single-BCR B cells from the SLE and HC groups. D. GSEA pathway enrichment analysis of single-BCR B cells from the SLE group compared to the HC group. (****p＜0.001)

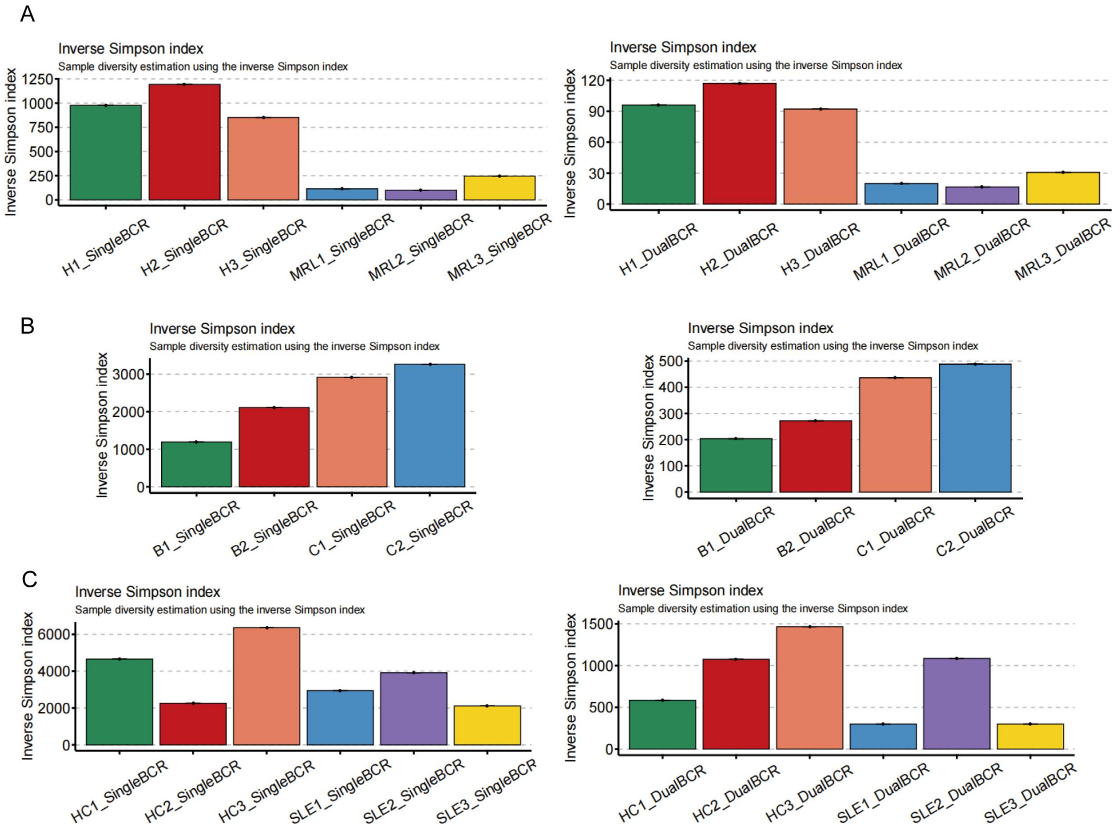

Sup Fig S12. Diversity analysis (inverse Simpson index) of single- and dual-BCR B cells across groups by integrated scRNA-seq and scBCR-seq.

A. Diversity analysis of single- and dual-BCR B cells for individual samples in the MRL and H mouse model groups.B. Diversity analysis of single- and dual-BCR B cells for individual samples in the B and C mouse model groups.C. Diversity analysis of single- and dual-BCR B cells for individual samples in the SLE patient and HC volunteer groups.

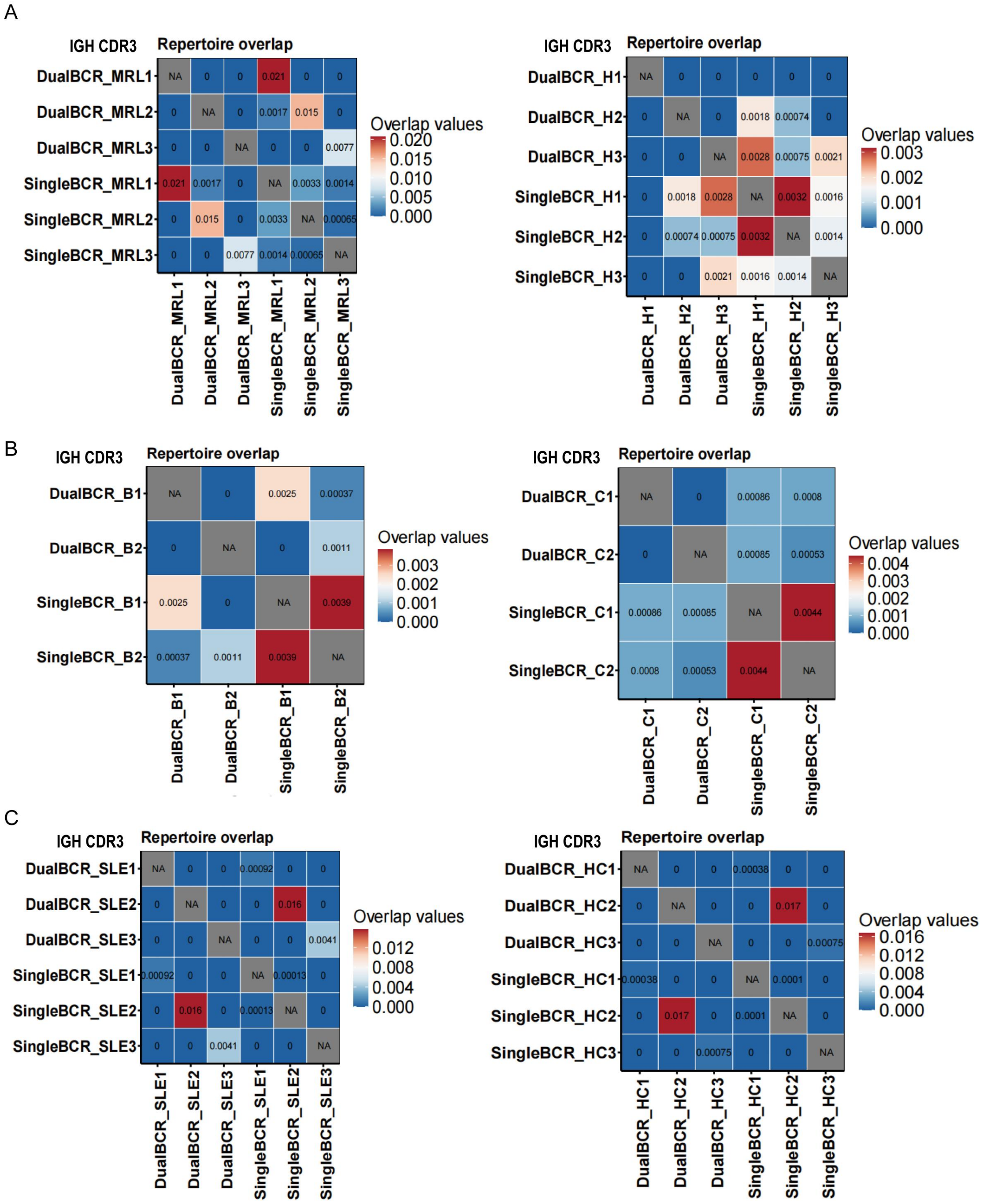

Sup Fig S13. Jaccard analysis of clonal overlap for single- and dual-BCR B cells across groups by integrated scRNA-seq and scBCR-seq.

A. Jaccard analysis of clonal overlap for single- and dual-BCR B cells across individual samples in the MRL and H mouse model groups.B. Jaccard analysis of clonal overlap for single- and dual-BCR B cells across individual samples in the B and C mouse model groups.C. Jaccard analysis of clonal overlap for single- and dual-BCR B cells across individual samples in the SLE patient and HC volunteer groups.

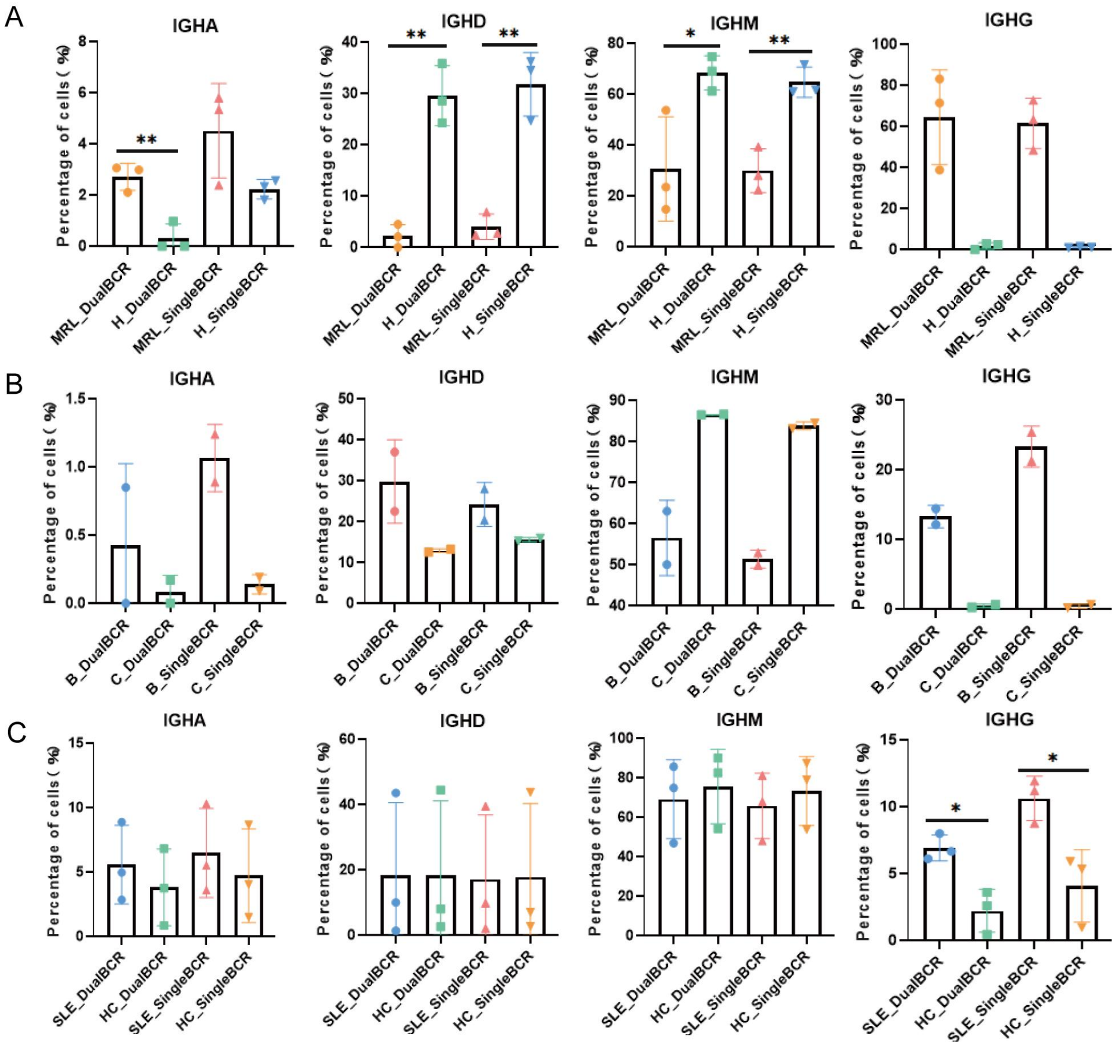

Sup Fig S14. Integrated scRNA-seq and scBCR-seq comparative analysis of immunoglobulin heavy chain (IGHA/IGHD/IGHM/IGHG) isotype distribution in single- and dual-BCR B cells from two SLE mouse models (MRL/lpr, SLE.Yaa) and SLE patients versus healthy controls.

A. Isotype distribution analysis of immunoglobulin heavy chains (IGHA, IGHD, IGHM, IGHG) in single- and dual-BCR B cells from MRL/lpr model mice and healthy control mice.B. Isotype distribution analysis of immunoglobulin heavy chains (IGHA, IGHD, IGHM, IGHG) in single- and dual-BCR B cells from SLE.Yaa model mice and healthy control mice.C. Isotype distribution analysis of immunoglobulin heavy chains (IGHA, IGHD, IGHM, IGHG) in single- and dual-BCR B cells from SLE patients and healthy volunteers.(*$p < 0.05$， **$p < 0.01$.)
